# A Promising Strategy to Treat Neurodegenerative Diseases by SIRT3 Activation

**DOI:** 10.3390/ijms24021615

**Published:** 2023-01-13

**Authors:** Alpna Tyagi, Subbiah Pugazhenthi

**Affiliations:** 1 Rocky Mountain Regional VA Medical Center, Aurora, CO 80045, USA; 2 Department of Medicine, University of Colorado-Anschutz Medical Campus, Aurora, CO 80045, USA

**Keywords:** SIRT3, longevity, mitochondria, metabolism, oxidative stress, neurodegeneration, metabolic syndrome, Alzheimer’s disease

## Abstract

SIRT3, the primary mitochondrial deacetylase, regulates the functions of mitochondrial proteins including metabolic enzymes and respiratory chain components. Although SIRT3’s functions in peripheral tissues are well established, the significance of its downregulation in neurodegenerative diseases is beginning to emerge. SIRT3 plays a key role in brain energy metabolism and provides substrate flexibility to neurons. It also facilitates metabolic coupling between fuel substrate-producing tissues and fuel-consuming tissues. SIRT3 mediates the health benefits of lifestyle-based modifications such as calorie restriction and exercise. SIRT3 deficiency is associated with metabolic syndrome (MetS), a precondition for diseases including obesity, diabetes, and cardiovascular disease. The pure form of Alzheimer’s disease (AD) is rare, and it has been reported to coexist with these diseases in aging populations. SIRT3 downregulation leads to mitochondrial dysfunction, neuroinflammation, and inflammation, potentially triggering factors of AD pathogenesis. Recent studies have also suggested that SIRT3 may act through multiple pathways to reduce plaque formation in the AD brain. In this review, we give an overview of SIRT3’s roles in brain physiology and pathology and discuss several activators of SIRT3 that can be considered potential therapeutic agents for the treatment of dementia.

## 1. Introduction

Calorie restriction (CR) in young rats was first reported in 1917 to prolong life span [1]. A later study confirmed the beneficial effects of CR on longevity and aging-associated diseases [2]. Silent information regulation-2 (Sir2) was discovered as a longevity gene by CR studies in budding yeast *Saccharomyces cerevisiae* [3], followed by the identification and characterization of seven mammalian sirtuins (SIRT1-7) [4,5,6]. They are distinct in their subcellular localization with SIRT1, SIRT6, and SIRT7 being nuclear whereas SIRT3, SIRT4, and SIRT5 are mitochondrial and SIRT2 is cytosolic. These sirtuins play key roles in longevity, metabolism, and inflammation. SIRT3, the focus of this review, is highly expressed in the brain [7]. It plays a central role in mitochondrial metabolism as a deacetylase enzyme and requires nicotinamide adenine dinucleotide (NAD+) for its activity. SIRT3 is transcriptionally upregulated by exercise and CR [8,9] whereas a chronic high-fat diet (HFD) decreases SIRT3 levels and depletes NAD+ [10]. The brain consumes 20% of total body energy, although it comprises only 2% of body weight. Brain hypometabolism is one of the causes of cognitive dysfunction [11]. SIRT3’s role in providing substrate flexibility is critical because each brain cell type has preferences for different energy substrates [12]. SIRT3’s functions in the peripheral tissues indirectly affect the brain because it facilitates the generation of energy substrates for the brain, suppresses chronic inflammation, enhances antioxidant defense, and promotes mitophagy. Therefore, these diverse actions of SIRT3 make it a suitable candidate to target for the treatment of AD and other neurodegenerative diseases.

## 2. SIRT3 and Longevity

Sirtuins in general and SIRT3 in particular have been shown to be associated with longevity. CR, which is well known to extend lifespan, increases the levels of SIRT3, leading to SIRT3-mediated deacetylation of mitochondrial proteins involved in diverse pathways [13]. During CR in mice, SIRT3 upregulates glutathione-mediated mitochondrial antioxidant defense system, resulting in delayed progression of age-related hearing loss [14]. SIRT3 genotype variability is associated with aging. Specifically, elderly males with the TT genotype of SIRT3 were found to have an increased lifespan, whereas the GT genotype was associated with decreased survival [15]. Sirt3 gene variations were investigated in an Italian city’s elderly population and an association between longevity and both Sirt3 rs11555236 and rs4980329 was observed [16]. In an interesting study, Barger et al. analyzed gene expression data sets in multiple tissues of mice subjected to CR [17]. These signatures of delayed aging in this CR mouse model, in a tissue-independent manner, were found to be a common aspect of extended longevity. Mice lacking Sirt3, however, did not display these signatures. SIRT3’s action on delaying senescence has been studied at the cellular level. SIRT3 is enriched in hematopoietic stem cells (HSCs) where it modulates a stress response [18]. SIRT3 upregulation improves the regenerative capacity of aged HSCs, suggesting SIRT3’s ability to reverse aging-associated degeneration. Similarly, overexpression of Sirt3 in later-passage mesenchymal stem cells reduces aging-related senescence [19]. In contrast, accelerated senescence of human mesenchymal stem cells is observed following Sirt3 gene deletion [20]. Moreover, high glucose (HG)-induced cellular senescence in human diploid fibroblasts is prevented by Sirt3 overexpression [21].

## 3. Subcellular Localization

Subcellular localization of SIRT3 has been a subject of debate among earlier studies. Onyango et al. first reported that SIRT3 is an NAD+-dependent deacetylase and is localized to the mitochondria with a targeting N-terminal sequence [22]. While some studies have reported full-length SIRT3 localization to the nucleus, other studies suggest that SIRT3 is proteolytically modified and localized only to the mitochondria where it acts as an active protein deacetylase [23]. Full-length mouse SIRT3 protein localized to the mitochondria has no deacetylation activity in vitro unless it is truncated by proteolytic cleavage [24]. Using fluorescence microscopy, subcellular fractionation, and various SIRT3 expression constructs, Cooper et al. observed human SIRT3 to be localized exclusively in the mitochondria [25]; however, SIRT3 localization has been shown to change during stress conditions. Scher et al. reported that SIRT3 localizes both in mitochondria and nucleus under normal cellular growth conditions whereas induction of cellular stress with etoposide or UV irradiation triggers SIRT3 translocation from the nucleus to the mitochondria [26]. Similarly, under conditions of cellular stress, such as oxidative stress and UV irradiation, nuclear full-length SIRT3 is rapidly degraded via the ubiquitin–proteasome pathway while the mitochondrial processed form of SIRT3 is unaffected [27]. In addition, nuclear SIRT3 degradation results in downregulated expression of stress-related and nuclear-encoded mitochondrial genes, suggesting a function of SIRT3 in the nucleus. Other sirtuins can also regulate SIRT3 localization. Co-expression of SIRT3 and SIRT5 results in SIRT3 localization to the nucleus, supporting a functional role in the nucleus [28].

## 4. Indirect CNS Actions of SIRT3 through Peripheral Tissues

**Figure 1:**
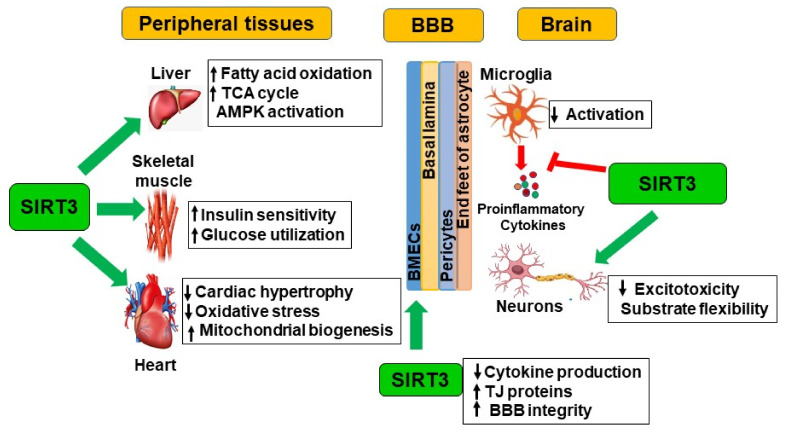
SIRT3 actions in multiple tissues: SIRT3 acts at the systemic level on multiple tissues including the liver, skeletal muscle, heart, BMECs, microglia, and neurons in the brain. It couples fuel-producing tissues with fuel-consuming tissues. SIRT3 provides metabolic flexibility to the tissues in terms of substrate utilization by activation of metabolic enzymes. While global silencing of Sirt3 leads to the acceleration of metabolic syndrome because of its broad systemic actions, tissue-specific knockdown of the Sirt3 gene does not result in dramatic changes in metabolic pathways. Beneficial actions are shown by green arrows and the inhibitory action is marked in red.

Although the main focus of this review is on the actions of SIRT3 in the brain, its effects in the peripheral tissues need to be discussed here because substrates for brain energy metabolism are generated in the periphery (Figure 1) [29,30]. SIRT3 deficiency-induced oxidative stress and inflammation at the systemic level can cause neuronal injury in the brain with circulating factors passing through the blood–brain barrier (BBB) [31]. In addition, comorbidities, including obesity, diabetes, and cardiovascular diseases often interact with AD pathogenesis and exacerbate it [32,33,34]. Several studies have demonstrated that brain lesions caused by comorbidities contribute to cognitive decline [35,36,37].

### 4.1. Liver

The liver plays a central role in the regulation of metabolic pathways. Both liver-specific Sirt3 overexpression in mice and in primary hepatocytes show an increase in oxygen consumption in isolated mitochondria as well as substrate utilization [38]. Liver-specific Sirt3 overexpression also facilitates oxidative metabolism [38]. Loss of Sirt3 in hepatocytes drastically impairs the regeneration process and causes mitochondrial dysfunction [39]. Under conditions of nutrient excess in mice, key mitochondrial proteins that protect against hepatic lipotoxicity are hyperacetylated, suggesting their downregulation [40]. Sirt3 deficiency accelerates HFD-induced nonalcoholic fatty liver disease in mice by impairing intestinal permeability through gut microbiota dysbiosis and inflammation [41]. Moreover, HFD feeding in ovariectomized Sirt3 knockout (Sirt3^−/−^) mice severely impacts liver metabolic parameters and promotes weight gain [42]. Mitochondrial dysfunction is observed in hepatocytes isolated from HFD-fed yellow fish and HepG2 cells exposed to fatty acid [43]. Dietary choline normalizes HFD-induced hepatic lipid dysregulation by increasing Sirt3 expression [43]. Sirt3 silencing in HepG2 cells leads to downregulated phosphorylation of AMP-activated protein kinase (AMPK) resulting in lipid accumulation [44]. Bao et al. have reported disruption in the mitochondrial electron transfer chain, reduction in mitochondrial membrane potential, and induction of reactive oxygen species in Sirt3-depleted HepG2 cells [45]. In contrast, Sirt3 overexpression reduces lipid accumulation via AMPK activation in human hepatic cells [44].

### 4.2. Skeletal Muscle

Skeletal muscle metabolism is closely linked to exercise, calorie utilization, and insulin sensitivity. Oxygen consumption in skeletal muscle decreases in Sirt3^−/−^ mice, leading to oxidative stress-induced JNK activation and defective insulin signaling [46]. The authors also confirmed these findings using Sirt3 gene silencing in cultured myoblasts. SIRT3 protein levels in skeletal muscle are elevated in response to exercise training and fasting, leading to phosphorylation of AMPK and cAMP response element-binding protein (CREB), resulting in the activation of peroxisome proliferator-activated receptor gamma coactivator-1alpha (PGC-1α), a target of SIRT3 [47]. Conversely, Sirt3-null animals show significantly less activation of AMPK, CREB, and PGC-1α expression in response to exercise training and fasting [47]. Silencing of Sirt3, in vivo and in cultured myoblasts, suppresses pyruvate dehydrogenase activity (PDH) resulting in the switching of substrate utilization from carbohydrate oxidation towards the lactate production and fatty acid utilization, even in the fed state [48]. Similarly, Lantier et al. have demonstrated the shifting of TCA cycle-based respiration towards fatty acid-oxidation in permeabilized muscle fibers of HFD-fed Sirt3^−/−^ mice because of decreased hexokinase 2 activity [49]. These findings suggest that SIRT3 is essential for glucose utilization. Moreover, insulin resistance is observed in Sirt3^−/−^ mice as shown by the hyperglycemic-euglycemic clamp experiment [49]. Both mitochondrial respiratory chain enzyme activity and antioxidant defense are decreased in the skeletal muscle of obese pregnant women with reduced SIRT3 activity [50].

### 4.3. Heart

The heart contains the highest content of mitochondria, occupying one-third of the cell volume in cardiac myocytes because of high energy demands. SIRT3 delays the progression of heart failure and cardiac hypertrophy by improving mitochondrial biogenesis and by reducing reactive oxygen species (ROS) cellular levels [51]. In this regard, studies have shown that Sirt3-deficient mice have signs of cardiac hypertrophy [51] and cardiac abnormalities due to defective trans-mitochondrial cristae alignment and impaired mitochondrial bioenergetics [52]. While Sirt3-expressing transgenic (Tg) mice are protected from cardiac hypertrophy [51], endothelial-specific Sirt3KO (ECKO) mice show myocardial capillary rarefaction along with reduced coronary flow reserve (CFR) and diastolic dysfunction [53]. Using a mouse cardiac hypertrophy model induced by transverse aortic constriction (TAC), Chen et al. have reported significantly higher abnormal lipid accumulation in the heart because of decreased fatty acid oxidation rates in Sirt3^−/−^ mice [54]. This finding was further supported by the higher acetylation levels of long-chain acyl CoA dehydrogenase (LCAD), a key enzyme in fatty acid oxidation [54]. In another study, Koentges et al. observed reduced cardiac function in isolated working hearts of Sirt3^−/−^ mice, due to decreased oxidation of palmitate and glucose along with reduced oxygen consumption rates [55]. Zeng et al. have shown cardiac hypertrophy and dysfunction with increased ROS production due to decreased Sirt3 expression in HFD-fed mice [56]. Additionally, HFD-fed Sirt3^−/−^ mice display obesity-related cardiac dysfunction, cardiac remodeling, and elevated cardiac inflammation via modulation of the ROS-NF-κB-MCP-1 pathway [57]. Conversely, moderate CR increases Sirt3 expression, leading to improved cardiac function in female Sprague-Dawley rats [58]. The mechanism of SIRT3 action involves forkhead box O3a (Foxo3a)-dependent induction of manganese superoxide dismutase (MnSOD) and catalase in primary cultured cardiomyocytes, resulting in decreased ROS cellular levels [51]. Another pathway involved in SIRT3-mediated improvement in mitochondrial biogenesis is through the AMPKα-PGC-1α axis [59].

### 4.4. Vascular Endothelial Cells

Endothelial cells (ECs) that line the blood vessels regulate blood flow, vascular permeability, and angiogenesis. Sirt3 expression is needed for maintaining vascular cell homeostasis and endothelial barrier integrity [60]. People with diabetes are more likely to develop cardiovascular disease and both diseases are known to cause vascular dementia, the second most common form of dementia, after AD [61]. Sirt3 gene deletion in mice promotes vascular dysfunction and hypertension [62]. Moreover, Sirt3 deficiency results in high mtROS levels which further contributes to vascular dysfunction in both obese mice and humans [63]. Global Sirt3 gene deletion leads to hyperacetylation of mitochondrial proteins, including superoxide dismutase (SOD2), and increased vascular permeability and vascular inflammation [64]. Conversely, transgenic mice with global Sirt3 overexpression are protected from endothelial dysfunction, vascular oxidative stress, and hypertrophy [64]. Sirt3-deficient mice fed a high-cholesterol diet exhibit impaired superoxide-dependent endothelial dysfunction [65]. Defective insulin-mediated vasorelaxation is observed in Sirt3-deficient mice fed a HFD [63]. Conversely, SIRT3 enhances insulin sensitivity in human umbilical vein endothelial cells (HUVEC) [63]. Patients with hypertension have decreased Sirt3 expression leading to hyperacetylation of mitochondrial proteins [62]. Circulating ECs (CD34+) from diabetic patients show a loss of SIRT3 expression and an associated decrease in cell viability [66]. Sirt3 gene silencing in ECs results in reduced hypoxia-induced expression of HIF-2α, VEGF, and Ang-1 [53]. Furthermore, Sirt3-silenced HUVECs exposed to HG show SOD inactivation and cytotoxicity [66]. Exposure of ECs to HG as in diabetes decreases Sirt3 expression, leading to increased senescence-associated galactosidase and loss of the ability to form tubular networks [67]. Sirt3 overexpression, on the other hand, protects HG-induced angiogenic dysfunction and results in decreased p53 acetylation, revealing a potential molecular mechanism involving HG, Sirt3, and P53 in endothelial cell senescence [67]. Moreover, Sirt3 activates the AMPK pathway, maintains redox balance, and inhibits the caspase-9-involved apoptosis pathway in HG-exposed endothelial cells [68].

### 4.5. Brain Microvascular Endothelial Cells

Brain microvascular endothelial cells (BMECs) are a critical component of BBB that protect the brain from the toxins generated in the periphery while allowing essential nutrients to pass through. BMECs along with pericytes and end feet of astrocytes form the neurovascular unit (NVU), the gateway between the periphery and the interior milieu of the brain. Because of this critical role, BMECs are tightly held together by tight junction proteins. We have reported a decrease in the levels of SIRT3 and tight junction proteins in the brain of APP/PS1 mice fed a western diet and in human post-mortem AD brain samples [69]. In this study, decreased levels of SIRT3 and tight junction proteins (claudin-5 and ZO-1) were observed in BEND3 cells, a mouse brain endothelial cell line exposed to a combination of HG and palmitic acid. This treatment also resulted in the induction of proinflammatory cytokines. Interestingly, this induction was more in Sirt3-silenced BEND3 cells. Another study reported that overexpression of Sirt3, in a human brain microvascular endothelial cells (HBMEC) I/R injury model, decreases the permeability of HBMECs and the growth of endothelial cells [70]. Furthermore, the beneficial actions of PPAR-γ in this injury model are mediated through the activation of SIRT3.

## 5. Pathways of SIRT3 Action

SIRT3 deacetylates and activates several mitochondrial proteins, its actions are seen in diverse pathways that are relevant in the pathogenesis of AD and other neurodegenerative diseases. Therefore, a therapeutic agent that targets multiple pathways related to AD will be a suitable candidate to prevent/delay AD pathogenesis because clinical trials targeting a single pathology have failed. Furthermore, many of these pathways are dysregulated during other neurodegenerative diseases as well. SIRT3 action in these pathways will be discussed in this section.

### 5.1. SIRT3 and Mitochondrial Bioenergetics

As the primary mitochondrial deacetylase, SIRT3 plays a central role in mitochondrial energy homeostasis [22]. Accumulation of acetyl-CoA, especially after calorie overload, leads to non-enzymatic acetylation at lysine residues of mitochondrial proteins, resulting in mitochondrial dysfunction, a process that is reversed by SIRT3 overexpression. Silencing of Sirt3 gene results in increased acetylation of multiple components of complex I of the electron transport chain which can be ameliorated by incubation of exogenous SIRT3 with mitochondria [71]. SIRT3 is essential for the maintenance of basal ATP levels and in the regulation of mitochondrial electron transport [71]. SIRT3-mediated deacetylation of ATP synthase proteins in the liver has been demonstrated by in vivo experiments in Sirt3^+/+^ and Sirt3^−/−^ mice and with cultured HEK 293T cells [72]. Mice lacking Sirt3 have significantly reduced basal levels of ATP in the heart, kidney, and liver with elevated mitochondrial protein acetylation in these tissues [71]. SIRT3 deacetylates the succinate dehydrogenase flavoprotein (SdhA) a complex II subunit and increases its activity [73]. The acetylated hydrophilic surface of SdhA regulates the entry of substrate into the active site of the protein and therefore controls succinate dehydrogenase activity [73]. Moreover, increased acetylation of MRPL10 in Sirt3^−/−^ mice results in the enhanced translational activity of mitochondrial ribosomes [74]. Similarly, Sirt3 silencing in C2C12 cells results in increased mitochondrial protein synthesis while ectopic expression of Sirt3 results in deacetylation of MRPL10 and suppression of protein synthesis in MRPL10 which maintains the basal oxidative phosphorylation. In fact, this negative effect of SIRT3 on mitochondrial protein synthesis is considered beneficial as it slows down the cellular aging process. Moreover, SIRT3 itself is hyperacetylated at Lys57, resulting in the decrease of its deacetylase activity [75].

### 5.2. Regulation of Brain Metabolism by SIRT3

The energy demands of the brain are very high especially with neurons consuming a major portion of the energy produced from the peripheral tissues. Furthermore, each brain cell type has preferences for different energy substrates [12]. For example, neurons produce energy by oxidative metabolism involving the TCA cycle, whereas glycolysis is predominant in astrocytes [12,76]. Astrocytes play a critical role in synaptic activity by delivering lactate to synapses. Thus, neurons and astrocytes are complementary in nature in terms of the metabolic pathways with astrocytes serving a supporting function [12]. In the case of microglia, its activation state causes a metabolic shift. Microglia which regulate the innate immune response in the brain constantly survey the brain parenchyma, making contacts with neuronal synapses and translocating to the injured sites for phagocytosis. Because of these functions, they are in constant demand for ATP [76]. Quiescent microglia and activated microglia meet their energy requirements by oxidative phosphorylation and glycolysis, respectively. Sirt3 deficiency is expected to dysregulate microglia through multiple mechanisms. First, peripheral inflammation is known to prime the microglia, leading to its exacerbated response to later stimulation [77]. Second, microglia are known to change their phenotype in response to stress in the brain environment, especially in neuronal synapses [78]. Third, Sirt3 downregulation within microglia can also affect its own metabolism [76]. The glycolytic pathway following microglial activation is known to drive pro-inflammatory phenotype whereas mitochondrial oxidative phosphorylation is anti-inflammatory in nature. When glycolytic products including G6P and G3P accumulate, and they feed into the pentose phosphate pathway to generate NADPH which is a substrate for NADPH oxidase, leading to ROS generation. Genome-wide association studies (GWAS) of AD patients have shown a large number of risk factor genes to be associated with microglia [79]. Post-translational modification of metabolic enzymes by lysine acetylation is a key regulatory mechanism, and SIRT3 is the primary mitochondrial deacetylase. Rardin et al. have identified targets of SIRT3 using a label-free mass spectrometry approach with the liver samples of Sirt3^−/−^ mice [80]. In the absence of SIRT3, lysine acetylation of 283 sites in 136 mitochondrial proteins was significantly increased, suggesting a role of SIRT3 in the regulation of multiple metabolic pathways including fatty acid oxidation, ketogenesis, amino acid catabolism, and the urea and tricarboxylic acid cycles. Dittenhafer-Reed et al. performed acetylome analysis with multiple tissues, including the brain, heart, kidney, liver, and skeletal muscle, of Sirt3^−/−^ mice and reported that SIRT3 regulates metabolic pathways differentially in fuel-producing and fuel-utilizing tissues [81]. The authors also provided evidence to show the utilization of ketone bodies in the brain. Following an acetylome analysis by label-free mass spectrometry and gene ontology pathway analysis, we reported downregulation of enzymes in fatty acid oxidation and the TCA cycle in the brain samples of Sirt3^−/−^ mice fed a western diet, a model of MetS [82]. Our study was the first to examine the brain acetylome in this model that combines genetic and lifestyle-based risk factors of MetS.

SIRT3 promotes an adaptive response to metabolic stress by providing metabolic substrate flexibility to fuel-consuming tissues. For example, SIRT3 promotes the urea cycle and β-oxidation during diet restriction by deacetylating and activating key enzymes in these pathways [83]. SIRT3 regulates fatty acid oxidation by deacetylating long-chain acyl-CoA dehydrogenase (LCAD) at residues Lys-318 and Lys-322 leading to its activation [84]. SIRT3 can regulate ketone body production in hepatic mitochondria by deacetylating 3-hydroxy-3-methylglutaryl CoA synthase 2 (HMGCS2), a rate-limiting step in ketogenesis [85]. These ketone bodies provide substrates for brain neurons during fasting.

### 5.3. Anti-Inflammatory Effects of SIRT3

As part of its central role in mitochondrial function, SIRT3 exerts anti-inflammatory effects in response to mitochondrial injury, which is known to result in inflammasome formation and triggering the inflammatory pathway [86,87]. Calorie overload is known to induce NLRP3 inflammasome formation through the disruption of mitochondrial integrity [88]. Conversely, prolonged nutrient deprivation reduces NLRP3 inflammasome formation in wild-type but not in Sirt3^−/−^ mice [89]. The negative regulatory effect of Sirt3 on NLRP3 is due to the deacetylation of mitochondrial SOD2, leading to SOD2 activation. We observed inflammasome formation leading to elevated IL-1β expression in the brains of Sirt3^−/−^ mice which were further exacerbated following western diet feeding [82]. There were also elevated circulating C-reactive protein levels in these mice suggesting peripheral inflammation. We observed exacerbated microglial activation and elevated levels of IL-1β, TNF-α, and Cox-2 in the brain of APP/PS1/Sirt3^−/−^ mice, a comorbid Alzheimer’s mouse model generated by our lab [90]. While SIRT3 overexpression in neural stem cells (NSCs) reduces microglia cellular damage, and its knockdown via siRNA promotes cellular injury. Intermittent fasting, which is known to elevate SIRT3 levels, decreases neuroinflammation, and it is attenuated in mice with microglia-specific Sirt3 gene deletion [91]. This study also suggested the activation of the Nrf2/HO-1 pathway by SIRT3 as a possible mechanism. We have reported microglial proliferation in the brains of Sirt3^−/−^ mice fed a western diet [82].

### 5.4. Antioxidant Effects of SIRT3

SIRT3 has been shown to reduce mitochondrial ROS generation in multiple cell types. SIRT3 deacetylates two lysine residues on superoxide dismutase 2 (SOD2; MnSOD) and increases its antioxidative activity [92]. This action during CR enhances SOD2’s ability to reduce cellular ROS levels. The brain is vulnerable to oxidative stress because of the low-level expression of antioxidant enzymes compared to other tissues. We have identified several antioxidant enzymes including SOD2 that were downregulated in the brain of a mouse model of MetS [82]. Using an aging rat model, Zeng et al. have demonstrated that age-associated reduction in SIRT3 expression in the auditory cortex is associated with increased SOD2 acetylation, leading to its reduced activity, and the accumulation of ROS [93]. The antioxidant effects of hydrogen sulfide in vascular endothelial cells have been shown to involve the induction of Sirt3 at the promoter level [94]. SIRT3 regulates mitochondrial redox states by deacetylation and activation of isocitrate dehydrogenase 2 (IDH2), the major source of NADPH [95]. SIRT3 also increases the expression of antioxidant enzymes, such as MnSOD and catalases by activation of the transcription factor Foxo3a [51]. Similarly, SIRT3 protects mitochondria against oxidative stress by deacetylation of FOXO3 at K271 and K290, resulting in its activation leading to the upregulation of genes involved in mitochondrial homeostasis, modulation of ATP production, and clearance of defective mitochondria in endothelial cells [96]. Thus, SIRT3 plays a dual role in reducing ROS levels by increasing the expression as well as the activation of antioxidant enzymes.

### 5.5. Mitophagy and Autophagy

Autophagy is a lysosomal degradation pathway, and mitophagy is the autophagic turnover of damaged mitochondria. Mitophagy is regulated by a signaling pathway involving phosphatase and tensin homolog (PTEN)-induced putative kinase 1 (PINK1) and Parkin [97]. Sirt3 has been shown to activate mitophagy through this Foxo3a/PINK1-Parkin pathway [98]. SIRT3/Foxo3A/Parkin-mediated mitophagy is downregulated in diabetic cardiomyocytes which is further aggravated in Sirt3 KO mice [99]. Advanced glycation end products (AGEs) accumulation has been shown to promote bone marrow mesenchymal stem cells (BMSCs) senescence leading to senile osteoporosis (SOP) by a mechanism involving reduced SIRT3 expression and inhibition of mitophagy [100]. This study also demonstrated that overexpression of Sirt3 in senescence-accelerated mouse strain P6 (SAMP6) by administration of recombinant adeno-associated virus 9 carrying Sirt3 plasmid alleviates BMSCs senescence and the formation of SOP [101]. Furthermore, SIRT3 has been also shown to activate autophagy in several cell types. It protects against sepsis-induced small-intestine injury by a mechanism involving the induction of autophagy [102]. Overexpression of Sirt3 in adipocytes activates autophagy on lipid droplets resulting in reduced lipid accumulation [103]. Sirt3 inhibits Angiotensin II-induced myocardial hypertrophy by activation of autophagy [104]. Stable overexpression of Sirt3 in SH-SY5Y cells protects a rotenone-induced injury through the regulation of autophagy [105]. Decreases in the levels of autophagic components including Beclin-1 and LC-3II have observed in HG-exposed HK-2 cells, a human renal tubular epithelial cell line, and overexpression of Sirt3 restores autophagy via downregulation of Notch-1/Hes-1 [106]. The adipokine omentin improves myocardial infarction-induced heart failure by activating mitophagy via the SIRT3/FOXO3 pathway [101]. Mitochonic acid-5 (MA-5) activates SIRT3 and promotes Parkin-dependent mitophagy in chondrocytes whereas 3-(1H-1,2,3-triazol-4-yl) pyridine (3-TYP), a specific inhibitor of SIRT3, blocks the SIRT3/Parkin pathway [107]. Thus, SIRT3’s protective actions in several injury models involve mitochondrial quality control by mitophagy.

## 6. SIRT3 Downregulation in Disease Conditions

### 6.1. Metabolic Syndrome and Diabetes

SIRT3 downregulation is a critical component of MetS because global Sirt3 deletion accelerates the development of MetS, single nucleotide polymorphism of human SIRT3 is associated with susceptibility for MetS and calorie overload depletes NAD+, a coenzyme needed for SIRT3 activity [10]. Sirt3 gene deletion causes insulin resistance as shown by the hyperinsulinemic-euglycemic clamp technique, and defective glucose uptake in skeletal muscle [49]. This study also reported that there is a substrate switch from glucose to fatty acids in muscle. We performed acetylome analysis with mitochondria isolated from the brain samples of western diet-fed Sirt3^−/−^ mice, a model that combines genetic and lifestyle risk factors of MetS and identified 103 hyperacetylated proteins suggesting their downregulation [82]. GO pathway analysis identified four major pathways, namely, the electron transport chain, fatty acid oxidation, the TCA cycle, and the redox pathway to be downregulated. The role of dysregulated SIRT3 pathways during diabetic complications have been discussed in the preceding sections.

### 6.2. Role of SIRT3 Downregulation in Neurodegenerative Diseases

SIRT3 plays significant roles in brain health by regulating directly metabolic pathways in different brain cell types and by its indirect actions in peripheral tissues. The neuroprotective effects of SIRT3 by reducing oxidative and inflammatory injuries have been also reported by several studies. Sirt3 overexpression protects cultured mouse primary cortical neurons from NMDA-induced excitotoxic injury [108]. Using a microfluidic culture device, Magnifico et al. reported that activation of SIRT3 by NAD+ prevents axonal degeneration and caspase activation in CNS primary neurons exposed to proapoptotic stimuli [109]. In primary cultured rat cortical neurons, Sirt3 silencing aggravates H_2_O_2_-induced oxidative neuronal injury, while its overexpression inhibits H_2_O_2_-induced neuronal injury by promoting endogenous antioxidant enzyme activity and mitochondrial biogenesis [110]. PGC-1α induces the Sirt3 promoter through estrogen-related receptor alpha (ERRα) in dopaminergic neurons and protects them from 1-methyl-4-phenyl-1,2,3,6 tetrahydropyridine (MPTP) [111]. SIRT3 is upregulated in microglia, astrocytes, and oligodendrocytes following hypoxic stress as a protective response [112]. Microglial activation-induced oxidative stress injury on NSCs has been shown to be prevented by SIRT3 in a coculture system [113]. Microglial activation-induced cytotoxicity in dopaminergic neuronal cells is alleviated by Sirt3 overexpression while Sirt3 gene aggravates neurotoxicity [114]. HIV TAT protein induces senescence in mouse primary microglial cells with downregulation of SIRT3 while overexpression of Sirt3 in these cells reverses this senescence-like phenotype [115]. Because of its role in brain homeostasis, several studies have reported SIRT3 downregulation in the following neurodegenerative diseases.

#### 6.2.1. Alzheimer’s Disease

The major hallmarks of AD are amyloid plaque deposition and neurofibrillary tangle formation [116]. Multiple other factors including neuroinflammation and mitochondrial dysfunction also play significant roles in Alzheimer’s pathogenesis [117,118]. Clinical trials targeting individual downstream pathologies have failed because of the complex nature of this disease. There is a need to target an upstream pathway during the cellular phase of AD. SIRT3 downregulation may be a potential trigger in that pathway. Studies have shown that both Sirt3 mRNA and SIRT3 protein levels are significantly lower in the cortex of APP/PS1 mice [119] and in human AD post-mortem samples [120]. However, Weir et al. reported that Sirt3 expression increases in parallel to β-amyloid deposition in a spatiotemporal manner in an AD mouse model [121]. This study also reported the induction of Sirt3 expression in cultured primary hippocampal neurons in response to oxidative stress. It is possible that Sirt3 upregulation may be an early protective response against neuroinflammation and oxidative stress but may not be sustained during Alzheimer’s pathogenesis. 

**Figure 2:**
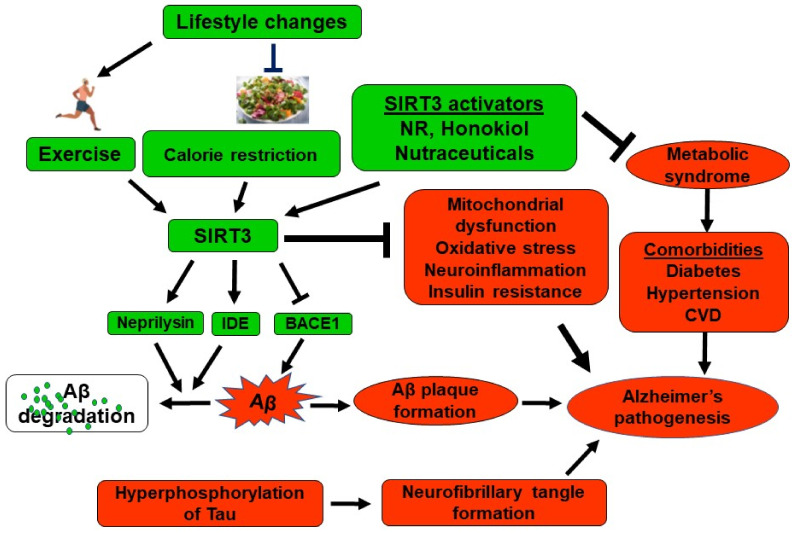
Protective effects of SIRT3 in Alzheimer’s disease: SIRT3 reduces the risk factors of Alzheimer’s pathogenesis by acting through multiple pathways. While SIRT3’s actions on alleviating mitochondrial dysfunction, oxidative stress, neuroinflammation, and insulin resistance are well known, recent studies are suggesting that it can also reduce amyloid plaque deposition by regulating the Aβ generation. We have recently demonstrated SIRT3-mediated decrease in BACE1, a key enzyme in the generation of Aβ peptide and increases in the levels of Aβ degrading enzymes, insulin degrading enzyme (IDE), and neprilysin. SIRT3 activators can ameliorate metabolic syndrome, the precondition for diabetes, hypertension, and cardiovascular diseases (CVD) which can coexist with Alzheimer’s disease. Protective actions are shown in green, and the pathways of disease progression are shown in red.

SIRT3 deficiency has been shown to be associated with MetS, the precondition for diabetes, cardiovascular diseases, and obesity, collectively known as comorbidities, which can coexist with AD. Broad systemic changes associated with these diseases have been shown to initiate and accelerate the molecular events of AD pathogenesis [122]. We have proposed a model suggesting that MetS-induced changes in the brain can interact with the early cellular phase of AD in mid-life and contribute to disease progression (Figure 2). To investigate the converging pathways, mediated by MetS and amyloid pathology, we generated APP/PS1/Sirt3^−/−^ mice as a comorbid AD model with MetS [90]. As expected, the combination of MetS and amyloid pathology resulted in an accelerated phenotype. There was more amyloid plaque deposition, insulin resistance, and microglial activation in APP/PS1/Sirt3^−/−^ mice, compared to APP/PS1 mice. In a similar study, Sirt3 haploinsufficiency in AD mice (Sirt3+/−AppPs1) has been shown to exacerbate the loss of GABAergic neurons and neuronal network hyperexcitability [123]. Sirt3 overexpression, on the other hand, rescues GABAergic interneurons and protects cerebral circuits against hyperexcitability. There are also other reports suggesting the potential of SIRT3 upregulation in improving cognitive function. Intermittent fasting, which is known to increase SIRT3 levels, enhances GABAergic tone, reduces anxiety-like behaviors, and improves hippocampus-dependent memory in wild type but not in SIRT3-deficient mice [124]. The neurotrophin pituitary adenylate cyclase-activating polypeptide (PACAP) protects cultured primary cortical neurons against Aβ-induced apoptosis, by a mechanism involving Sirt3 upregulation [125].

Several studies have suggested that SIRT3 may have a direct role in reducing amyloid plaque deposition in addition to interacting with AD through comorbidities. BACE1, a key regulatory enzyme in the generation of Aβ peptide is decreased following the administration of honokiol, an activator of SIRT3 [126]. Another interesting report speculates SIRT3-mediated BACE1 inhibition through LKB1, AMPK, CREB, PGC-1α, and PPARɣ using network analysis of SIRT3 binding substrates [127]. We identified decreased expression of IDE in Sirt3^−/−^ and APP/PS1/Sirt3^−/−^ mice, following RNA-seq analysis of the brain samples [128]. We observed in this study, increases in the levels of Aβ degrading enzymes, neprilysin, and insulin-degrading enzyme (IDE) in C57BL/6 mice treated with nicotinamide riboside (NR) which activates SIRT3. IDE promoter is known to be induced by the transcription factor Nrf1 [129] which in turn is induced by PGC-1α. Because SIRT3 activates PGC-1α, we speculate a potential mechanism through SIRT3/PGC-1α/Nrf1/IDE. This is supported by the fact that exercise, a known inducer of Sirt3 [9] is also reported to increase IDE levels in the liver [130]. The multiple pathways through which SIRT3 can decrease amyloid plaque deposition are presented in Figure 2. Indeed, we observed increased plaque deposition in the brain of APP/PS1/Sirt3^−/−^ mice compared to the APP/PS1 mouse brain [90]. However, further studies are needed to investigate SIRT3-mediated induction of IDE at the transcriptional level.

#### 6.2.2. Parkinson’s Disease

Oxidative stress and mitochondrial dysfunction are known to accelerate the pathogenesis of Parkinson’s disease (PD). SIRT3 has been shown to be protective in PD by acting through the mitochondrial antioxidant defense system [131]. 1-methyl-4-phenyl-1,2,3,6 tetrahydropyridine (MPTP) treatment is generally used to induce PD symptoms in both in vivo and in vitro models as it leads to the destruction of dopaminergic neurons. MPTP administration in the midbrain of mice leads to loss of PGC-1α and Sirt3, acetylation of SOD2 and ATP synthase β, and the loss of dopaminergic neurons is more severe in Sirt3^−/−^ mice [111]. Moreover, genetic ablation of Sirt3 amplifies oxidative stress and reduces mitochondrial membrane potential in substantia nigra pars compacta dopaminergic neurons [132]. Furthermore, increased acetylation of MnSOD at K68, a marker for its decreased activity, has been observed in PD post-mortem samples. Overexpression of Sirt3 alleviates oxidative stress-induced mitochondrial dysfunction and cell death in dopaminergic neurons and astrocytes [133]. Cui et al. reported acetylation of citrate synthase, a key mitochondrial enzyme, leading to a decrease in its activity in MPP+-treated SH-SY5Y cells, a human neuroblastoma cell line, [134]. SIRT3 overexpression in these cells results in the restoration of citrate synthase activity. Another study reported that SIRT3 overexpression protects rotenone-treated SH-SY5Y cells by decreasing apoptosis and ROS and by preventing α-synuclein accumulation whereas Sirt3 silencing results in increased cell apoptosis, reduced SOD and GSH, and increased accumulation of α-synuclein [135]. The activated BV2 cells, a mouse microglial cell line, have been shown to promote apoptosis and decrease Sirt3 expression in MN9D cells, mouse midbrain dopaminergic cell line [136] whereas Sirt3 expression in MN9D cells shows a neuroprotective effect against microglia activation-induced neuronal injury, by modulating mitophagy-NLRP3 inflammasome pathway [114].

#### 6.2.3. Huntington Disease

Huntington disease (HD) is a neurodegenerative disorder caused by an abnormal polyglutamine expansion within the N-terminal domain of the huntingtin protein (Htt). Mitochondrial dysfunction plays a key role in Htt-induced neurotoxicity. SIRT3 levels are less in cells expressing Htt [137]. Salamon et al. reported the elevation of Sirt3 mRNA in striatal, cortical, and cerebellar neurons as a protective response using N171-82Q transgenic mice as a model of HD [138]. While Sirt3 deficiency aggravates HD oxidative phenotype, its overexpression in cells expressing mutant HTT exerts antioxidant effects leading to improved mitochondrial functions. Interestingly, overexpression of SIRT3 fly-ortholog dSirt2 in HD flies reduces neurodegeneration and extends their life expectancy [139].

#### 6.2.4. Amyotrophic Lateral Sclerosis

Mutations in the Cu/Zn SOD1 gene are known to cause amyotrophic lateral sclerosis (ALS) with motor neuron loss. Fewer studies are listed in the literature on the contribution of Sirt3 in HD. Using cultured rat spinal cord motor neurons Song et al. reported that mutant SOD1^G93A^ expression by transfection causes a significant decrease in both mitochondrial and neurite length with an accumulation of round fragmented mitochondria and these effects by the mutant SOD1^G93A^ are rescued by both Sirt3 and PGC-1α expression [140]. Sirt3 activation by NR and small molecules has been shown to correct the defective metabolic profiles in induced pluripotent stem cells from familial and sporadic ALS patients [141].

#### 6.2.5. Ischemic Stroke and Other Brain Injury Models

Blood–brain barrier (BBB) is essential for maintaining the homeostasis of the brain microenvironment by protecting it from toxins generated in the periphery and by regulating the molecular exchange between blood and the brain. Sirt3 contributes to the maintenance of BBB integrity by its action on BMECs, especially the tight junction proteins. We have demonstrated that transendothelial electrical resistance is decreased with the co-cultures of Sirt3-silenced BEND3 and Sirt3-silenced BV2 cells [69]. Middle cerebral artery occlusion (MCAO)-induced ischemic stroke has been shown to be exacerbated in Sirt3^−/−^ mice [142]. The neurovascular recovery is also impaired in these mice. Using the same model of ischemic stroke, another study reported the protective effects of adjudin, a Sirt3 activator [143]. This contraceptive drug-mediated a decrease in glial scar area in wild type but not in Sirt3^−/−^ mice. Cerebral ischemia depletes brain tissue NAD+, an essential coenzyme for SIRT3 activity, and administration of nicotinamide mononucleotide (NMN), a precursor of NAD+ protects the brain from ischemic insult [144]. The mechanism involved is SIRT3 mediated normalization of hippocampal NAD+ pool in the mitochondria and SOD2 activity leading to reduction in ROS production. Cerebral ischemia-reperfusion injury has been shown to be associated with Sirt3 downregulation and mitochondrial fission [145]. This study also demonstrated amelioration of pro-survival signals, in neurons subjected to ischemia-reperfusion injury, following Sirt3 overexpression via the Wnt/β-catenin pathway. Another study reported the protective effects of melatonin in diabetic mice from cerebral ischemia-reperfusion injury via the Akt/SIRT3/SOD2 pathway leading to improvement in the infarct volume and neurological deficit [146]. Furthermore, the authors made similar observations in HT22 cells, a mouse hippocampal neuronal cell line exposed to HG which were inhibited by Sirt3 silencing and 3-TYP, a selective inhibitor of SIRT3. The protective effects of perampanel, a non-competitive inhibitor of AMPA receptor, were examined in a co-culture model of neurovascular unit consisting of neurons, astrocytes, and BMECs [147]. The authors observed perampanel mediated increase in SIRT3 protein levels and reduced protective effects with Sirt3 gene silencing. Furthermore, this inhibitor has been shown to preserve BBB function in vivo after traumatic brain injury (TBI) by increasing Sirt3 expression. Similarly, another study reported that adiponectin’s protective action post-TBI involves the restoration of SIRT3-mediated mitochondrial homeostasis [148]. Decreased Sirt3 expression is reported during subarachnoid hemorrhage [149] and lipopolysaccharide (LPS)-induced sepsis [150]. Sirt3 silencing leads to decreased expression of Tie-2 and HIF-2α/Notch3, resulting in increased vascular leakage, while Sirt3 overexpression by adenoviral Sirt3 prevents LPS-induced vascular leakage via the HIF-2α/Notch3 pathway [150].

## 7. Translational Significance of SIRT3 Activation

SIRT3, the mitochondrial deacetylase, regulates metabolism, antioxidant defense, and mitochondrial quality control. These actions have been demonstrated in multiple tissues. SIRT3 downregulation plays a significant role in several aging-associated diseases. Therefore, we discuss the therapeutic potential of SIRT3 activators in the treatment of these conditions. 

### 7.1. SIRT3 Activation by Lifestyle-Based Intervention

SIRT3 plays a major role in the health benefits of lifestyle changes including exercise and diet control [151]. These are also modifiable risk factors of AD. Multiple evidence shows that diet plays a pervasive role in shaping human health throughout life. Calorie overload by consumption of the western diet, or HFD leads to depletion of cellular NAD+ content and downregulation of SIRT3 expression and its activity resulting in MetS in one-third of the adult population in developed countries [10]. CR, on the other hand, activates SIRT3 by increasing NAD+ levels, [8,9] and extends lifespan as shown in a variety of species [152]. CR in young rats was first reported to prolong life span as early as in 1917 [1]. A later study confirmed the health benefits of CR on longevity and aging-associated diseases [2]. The positive effects of CR are seen particularly in delaying aging-associated diseases including AD. Mitochondria, the center of energy metabolism, act as a mediator of the health benefits of CR with SIRT3 playing a key role. Regular exercise is also a known component of a healthy lifestyle that is known to reduce the risk of diabetes and cardiovascular diseases, whereas its benefits on brain health and cognition were realized later. Mice subjected to running wheel exercises display increased Sirt3 expression in hippocampal neurons [9]. Conversely, HFD feeding of Sirt3^−/−^ mice results in cognitive dysfunction which is restored to normal by aerobic interval training via the SIRT3-MnSOD pathway [153]. A statistical study of Spearman’s correlations shows that middle-aged rugby players maintain brain resilience, memory, and executive functions, and high expression of peripheral gene expression of SIRT1 and SIRT3 [154]. The effects of exercise on the Sirt1–Sirt3 axis appear to play a significant role in the anti-aging resilience of the brain in these players. Another study has reported enhanced SIRT3 expression, accompanied by CREB phosphorylation and PGC-1α upregulation in skeletal muscle during exercise training [47]. Brandauer et al. observed a correlation of exercise training with SIRT3 and MnSOD protein levels in the quadriceps muscle of wild type but not in AMPK α2 kinase dead mice [155]. The authors also reported a reduction in SIRT3 and MnSOD in sedentary PGC-1α KO mice and increased MnSOD in the skeletal muscle of exercise-trained healthy humans. Thus, SIRT3 provides a molecular mechanism for healthy lifestyle habits.

### 7.2. NAD+ Precursors

In addition to diet, enzymes involved in the generation and breakdown of NAD+ can also regulate its levels. For example, along with metabolic decline during aging, the cellular NAD+ content decreases with elevated CD38, an enzyme that degrades NAD+ [156]. This study demonstrated the preservation of SIRT3 activity, NAD+ levels, and mitochondrial function in aged CD38 knockout mice. Conversely, overexpression of nicotinamide mononucleotide adenylyl transferase 3 (NMNAT3), in isolated bone marrow mesenchymal stem cells from rabbits, increases the level of NAD+ leading to improvement in SIRT3 activity, mitochondrial function, and resistance to stress-induced apoptosis [157]. Direct administration of NAD+ is not a viable therapeutic option because of its plasma instability and cell impermeability. To increase NAD+ cellular levels in vivo, generally its precursors NMN and NR have been administered. Klimova et al. have demonstrated an increase in the NAD+ pool of hippocampal mitochondria with a single dose administration of NMN [144]. Administration of NR prevents noise-induced hearing loss and spiral ganglia neurite degeneration [158]. These protective effects are not observed in Sirt3^−/−^ mice. Cognitive function has been shown to improve in Tg2576 mice, an Alzheimer’s mouse model following a 3-month treatment with NR [159]. In the brain of these treated mice, increases in the levels of NAD+, PGC-1α expression, BACE1 degradation, and the reduction in Aβ production were reported. Moreover, improvement in the long-term potentiation was observed in hippocampal slices from Tg2576 mice after exposure to NR.

The effects of NR have been also examined in human subjects. Chronic oral administration of NR (100 mg/day) is well tolerated in middle-aged and older humans [160]. This dose is also effective in stimulating NAD+ metabolism and in reducing systolic blood pressure. Another study tested the effects of higher doses of NR (300 and 1000 mg) orally for 8 weeks in overweight healthy women and men [161]. This treatment resulted in higher blood levels of NAD+ (51% and 142%) and its metabolites after 2 weeks of NR administration. Importantly, no significant side effects were observed in the NR-treated group. Raising NAD+ levels can also activate SIRT1, another key member of the sirtuin family. Zhang et al. have demonstrated the rejuvenation of muscle stem cells in aged mice through the NAD+/SIRT1 pathway [162]. SIRT3 itself is hyperacetylated at Lys57, resulting in the decrease in its deacetylase activity and SIRT1 restores SIRT3 function by deacetylation [75]. Therefore, NMN and NR can be expected to have broad-spectrum effects in the treatment of neurodegenerative diseases by activating these sirtuins.

### 7.3. Naturally Occurring SIRT3 Activators

Plant-based bioactive compounds have been studied worldwide by biomedical researchers for developing safe and effective drugs to prevent or treat various diseases. Recently, there is increased attention for using naturally occurring neuroprotective agents to treat chronic aging- associated diseases. Such molecules reported to activate SIRT3 are summarized in Table 1.

#### 7.3.1. Honokiol

Honokiol, a polyphenol originally isolated from the bark, seeds, and leaves of trees that belong to the genus *Magnolia,* can effectively pass through BBB [163] and therefore its therapeutic potential for neuromodulating effects have been studied [164]. Honokiol restores mitochondrial dysfunction and improves the cognitive deficits in PS1V97L Alzheimer’s transgenic mice [165]. BACE1, a key regulatory enzyme in the generation of Aβ peptide, is decreased by honokiol [126]. Hippocampal neuronal injuries caused by oxygen-glucose deprivation/reperfusion OGD/R are moderately rescued by honokiol treatment via SIRT3 activation [166]. Honokiol also ameliorates surgery-induced memory loss and decreases Sirt3 expression and neuroinflammatory injury in the hippocampus [167]. These rescuing effects of honokiol are impaired by treatment with 3-TYP, a SIRT3 inhibitor [167]. Other studies have demonstrated honokiol-mediated SIRT3 activation in vivo in choline-deficient HFD-fed mice [168] and carbon tetrachloride (CCl4)-stimulated liver-damaged mice [169] and in cultured primary mouse cardiomyocytes [170] and AML12 hepatocytes [168]. Using a zebrafish model of radiation-induced brain injury and HT22 cells, Liao et al. observed the neuroprotective effects following honokiol treatment via suppression of ROS, TNF-α, IL-1β, and Cox2, and elevated Sirt3 expression [171].

#### 7.3.2. Resveratrol

Resveratrol is a polyphenol present in grapes and consumed through products made from grapes such as red wine and grape juice. Studies have reported the beneficial actions of resveratrol, predominantly in cardiovascular diseases. Resveratrol activates SIRT3, regulates the acetylation of TFAM, and preserves mitochondrial function in diabetic rat hearts [172]. Moreover, it prevents pulmonary arterial hypertension (PAH) by increasing Sirt3 expression, deacetylating cyclophilinD, and preventing mPTP opening in a monocrotaline (MC)-treated rat model [173]. HUVEC cells exposed to resveratrol display increased SIRT3 activity and reduced mtROS generation leading to upregulation of FoxO3A-mediated mitochondria-encoded gene expression of ATP6, CO1, Cytb, ND2, and ND5, thereby leading to increased complex I activity and ATP synthesis [174]. Resveratrol improves mitochondrial quality control by activation of the Sirt1/Sirt3-Mfn2-Parkin-PGC-1α pathway [175]. Resveratrol-mediated protection of an immortalized mouse hippocampal cell line against ER stress is dependent on Sirt3-induced autophagy as shown by Beclin-1 expression and LC3II/LC3I ratio [176]. The neuroprotective effects of viniferin, a derivative of resveratrol, in HD involve the activation of AMPK, and SIRT3 expression [137]. Polydatin, a natural precursor and glycosylated form of resveratrol, protects against LPS-induced endothelial barrier disruption in SIRT3-dependent manner both in wild-type mice and in cultured HUVECs [177]. Polydatin also enhances SIRT3 deacetylase activity leading to improvement in mitochondrial function in HUVECs. SIRT3-mediated deacetylation of SOD2 and cyclophilin D (CypD) is also enhanced by polydatin administration and its protective effects are lost in the presence of a SIRT3 inhibitor (3-TYP) [177].

#### 7.3.3. Other Naturally Occurring SIRT3 Activators

Govindarajulu et al. have reviewed the potential of several nutraceutical-based natural compounds that activate SIRT3 including curcumin, salidroside, phloretin, acacetin, spinacetin, theacrine, trilobatin, sesamin, sesamol, and dihydromyricetin for the treatment of neurodegenerative diseases [178]. Curcumin has been shown to improve cognition in APPTG mice and reverse Aβ-42-induced metabolic dysfunction in neurons by activation of SIRT3 [179]. Neonatal rat cardiomyocytes exposed to salidroside, a glucoside found in the plant *Rhodiola rosea*, leads to activation of mitochondrial SIRT3, AMPK/Akt, and PGC-1α/TFAM followed by improved mitochondrial function [180]. Another natural phenol, phloretin, a dihydrochalcone, decreases the lysine acetylation of MnSOD and restores its activity by promoting the expression of Sirt3 and the phosphorylation of AMPK (Thr172) in both HUVECs and mouse aortas [181]. Acacetin, a flavone, protects vascular ECs by preserving mitochondrial function via activating Sirt1/Sirt3/AMPK signals in streptozotocin (STZ)-induced diabetic ApoE^−/−^ mice model and in cultured HUVECs exposed to glucose [182]. Spinacetin, a flavonoid glycoside found in spinach, protects against doxorubicin-induced cardiotoxicity in mice and in cultured H9C2 cells through SIRT3/AMPK/mTOR signaling pathway [183]. Theacrine, a purine alkaloid found in Chinese tea has been shown to activate SIRT3 and restore mitochondrial functions in multiple animal models of PD, namely, 6-OHDA-treated rats, MPTP-treated mice and zebrafish [184]. Trilobatin, a plant-based sweetener, inhibits Aβ (25-35) action, activates Sirt3/SOD2 signaling and reduces neuroinflammation both in vivo (APP/PS1 transgenic mice) and in vitro (Aβ_25–35_-treated BV2 cells) models [185]. The anti-apoptotic action of sesamin and sesamol, compounds derived from sesame seeds, involves the activation of SIRT1 and SIRT3 [186]. Administration of dihydromyricetin (DM), a flavanonol isolated from the Japanese raisin tree has been shown to reverse hypobaric hypoxia-induced cognitive dysfunction in adult rats by stimulating mitochondrial biogenesis and lowering lipid peroxidation in the hippocampus [187]. With studies in HT22 cells, the authors also showed that DM acts by a mechanism involving SIRT3-mediated FOXO3 deacetylation. The cardioprotective effects of elabela, a hormonal peptide that acts via the apelin receptor, in diabetic mice are dependent on SIRT3-mediated Foxo3a deacetylation [188]. Metformin, a synthetic derivative of a natural compound galegine, improves H9c2 myoblasts viability following myocardial hypoxia/reoxygenation (H/R) by activation of SIRT3 signaling pathway leading to SOD2 mediated reduction in ROS levels [189], This antidiabetic drug also protects salt-induced cardiovascular damage in mice by activating AMPK and by reducing the H3K27ac level on SIRT3 promoter [190].

**Table 1:** Naturally occurring SIRT3 activators.

Activators of Sirt3	Source	Disease Condition/Cell Type	References
Nicotinamide riboside chloride	Vitamin B3 derived	Hearing loss/ear Liver regeneration/	Brown et al. [158]
		AD	Gong et al. [159]
		Healthy Adults	Martens et al. [160]
		Healthy Overweight Adults	Conze et al. [161]
Honokiol	Magnolia	Glioma	Wang et al. [163]
		AD/neurons	Li et al. [165]
		Oxygen-Glucose Deprivation/Reperfusion-Induced Neuronal injuries/Neurons	Wan et al. [166]
		Memory loss/brain	Ye et al. [167]
		Liver damage/AML12 hepatocytes	Liu et al. [168]
		Liver damage/liver/AML12 hepatocytes	Liu et al. [169]
		Cardiac hypertrophy/primary cardiomyocytes	Pillai et al. [170]
		Radiation-induced brain injury	Liao et al. [171]
Resveratrol	Grapes, apples, peanuts, blueberries	Diabetic rat heart/heart/H9C2 cell line	Bagul et al. [172]
		Pulmonary arterialhypertension/cardiomyocytes	Bernal-Ramirez et al. [173]
		Cardiovascular/HUVECs	Zhou et al. [174]
		Myocardial Ischemia/Reperfusion Injury/ primary Rat cardiomyocyte	Zheng et al. [175]
		ER stress/HT22 cells-neuronal cell line	Yan et al. [176]
ε-Viniferin	Grapevines	Huntington disease/Primary cortical neurons	Fu et al. [137]
Polydatin	Grapes, peanuts, mulberry	Vascular endothelial/HUVECs Endothelial cells	Wu et al. [177]
Curcumin	Turmeric	Dementia/neurons	Liu et al. [149]
Salidroside	Rhodiola plants	Myocardial dysfunction/heart	Li et al. [180]
Phloretin	Apple and apricot	Cardiovascular/mouse aortas/HUVECs	Han et al. [181]
Acacetin	Safflower, snow lotus	Diabetic/aorta tissue/HUVECs	Han et al. [182]
Spinacetin	Spinach	Cardiotoxicity/ heart/H9C2 cell line	Liu et al. [183]
Theacrine	Camellia kucha	Parkinson’s disease/H-SY5Y cells	Duan et al. [184]
Trilobatin	Crabapple	Alzheimer’s disease/hippocampus /BV2 cells	Gao et al. [185]
		Injury in neurons/neuronal PC12	
Sesamin and sesamol	Fagara and Sesame oil	Neurodegenerative disorders/neuronal cells	Ruankham et al. [186]
Dihydromyricetin	Chinese vine tea plant	Memory impairment/ hippocampus/HT 22 cells	Liu et al. [187]
Elabela	Hormonal peptide	Diabetic	Li et al. [188]
Metformin	Galegine	Hypoxia/reoxygenation (H/R) injury/rat cardiomyocyte	Du et al. [189]
		Cardiovascular and liver diseases/heart and liver	Gao et al. [190]

## 8. Concluding Remarks

SIRT3, the primary mitochondrial deacetylase, is associated with longevity. It regulates the functions of several key mitochondrial proteins. Acetylome studies have identified the targets of SIRT3 action including metabolic enzymes and respiratory chain components. SIRT3 actions in multiple tissues have been investigated extensively by in vivo and in vitro studies. Its role in brain function is beginning to emerge. The energy consumption of brain is very high. SIRT3 facilitates the generation of energy substrates for the brain in the periphery and provides substrate flexibility to brain cell types. SIRT3 reduces cellular ROS levels via a dual mechanism of activation of antioxidant enzymes by deacetylation and by inducing their expression at the transcriptional level. The antioxidant effects of SIRT3 are critical for brain because it is vulnerable to oxidative stress-induced injury due to low level expression of antioxidant enzymes. By deacetylation of key mitochondrial proteins, SIRT3 ameliorates mitochondrial dysfunction, oxidative stress, and neuroinflammation, which are potential risk factors of Alzheimer’s pathogenesis. SIRT3 can also exert beneficial actions in diseases including diabetes and cardiovascular diseases which often coexist with AD. A growing body of evidence shows that SIRT3 downregulation contributes to aging-associated diseases including diabetes, cardiovascular diseases, and neurodegenerative diseases such as AD, PD, HD, and ALS. Restoring of its expression by lifestyle-based intervention, NAD+ precursors (NR) or by using naturally occurring compounds (honokiol, resveratrol etc.), is beneficial to delay/prevent age-related diseases. SIRT3 expression is increased by exercise and calorie restriction. In contrast, calorie overload decreases its levels and depletes NAD+ needed for its activation.

## Data Availability

Not applicable.

## Abbreviations

AGEs: advanced glycation end products; ALS, amyotrophic lateral sclerosis; AMPK, AMP-activated protein kinase; AD, Alzheimer’s Disease; BBB, blood brain barrier; BMSCs, bone marrow mesenchymal stem cells; BMECs, brain microvascular endothelial cells; CR, calorie restriction; CREB, cAMP response element-binding protein; CFR, Coronary flow reserve; DM, Dihydromyricetin; ECs, Endothelial cells; ECKO, Endothelial-specific Sirt3KO; FoxO3A, Forkhead box O3A; HSCs, hematopoietic stem cells; HBMEC, human brain microvascular endothelial cells; HD, Huntington disease; HG, high glucose; HFD, high-fat diet; HUVEC, human umbilical vein endothelial cells; IDE, insulin-degrading enzyme; IDH2, isocitrate dehydrogenase 2; LCAD, long-chain acyl CoA dehydrogenase; MnSOD, manganese superoxide dismutase; MetS, metabolic syndrome; MPTP, 1-methyl-4-phenyl-1,2,3,6 tetrahydropyridine; NVU, neurovascular unit; NMN, nicotinamide mononucleotide; NR, nicotinamide riboside; NSCs, neural stem cells; PD, Parkinson’s disease; PGC-1α, peroxisome proliferator-activated receptor gamma coactivator 1-alpha; PAH, pulmonary arterial hypertension; ROS, reactive oxygen species; SOP, senile osteoporosis; Sir2, Silent information regulation2; SIRT, Sirtuins; Sirt3^−/−^, Sirt3 knock out; SOD, superoxide dismutase; Tg, transgenic; TAC, transverse aortic constriction; TBI, traumatic brain injury.

## References

[1] Osborne T.B., Mendel L.B., Ferry E.L. (1917). The Effect of Retardation of Growth Upon the Breeding Period and Duration of Life of Rats. Science.

[2] Berg B.N., Simms H.S. (1960). Nutrition and longevity in the rat. II. Longevity and onset of disease with different levels of food intake. J. Nutr..

[3] Kaeberlein M., McVey M., Guarente L. (1999). The SIR2/3/4 complex and SIR2 alone promote longevity in Saccharomyces cerevisiae by two different mechanisms. Genes Dev..

[4] Yang Y.H., Chen Y.H., Zhang C.Y., Nimmakayalu M.A., Ward D.C., Weissman S. (2000). Cloning and characterization of two mouse genes with homology to the yeast Sir2 gene. Genomics.

[5] Su A.I., Wiltshire T., Batalov S., Lapp H., Ching K.A., Block D., Zhang J., Soden R., Hayakawa M., Kreiman G. (2004). A gene atlas of the mouse and human protein-encoding transcriptomes. Proc. Natl. Acad. Sci. USA.

[6] Michan S., Sinclair D. (2007). Sirtuins in mammals: Insights into their biological function. Biochem. J..

[7] Sidorova-Darmos E., Wither R.G., Shulyakova N., Fisher C., Ratnam M., Aarts M., Lilge L., Monnier P.P., Eubanks J.H. (2014). Differential expression of sirtuin family members in the developing, adult, and aged rat brain. Front. Aging Neurosci..

[8] Nogueiras R., Habegger K.M., Chaudhary N., Finan B., Banks A.S., Dietrich M.O., Horvath T.L., Sinclair D.A., Pfluger P.T., Tschop M.H. (2012). Sirtuin 1 and sirtuin 3: Physiological modulators of metabolism. Physiol. Rev..

[9] Cheng A., Yang Y., Zhou Y., Maharana C., Lu D., Peng W., Liu Y., Wan R., Marosi K., Misiak M. (2016). Mitochondrial SIRT3 Mediates Adaptive Responses of Neurons to Exercise and Metabolic and Excitatory Challenges. Cell Metab..

[10] Hirschey M.D., Shimazu T., Jing E., Grueter C.A., Collins A.M., Aouizerat B., Stancakova A., Goetzman E., Lam M.M., Schwer B. (2011). SIRT3 deficiency and mitochondrial protein hyperacetylation accelerate the development of the metabolic syndrome. Mol Cell.

[11] Cunnane S., Nugent S., Roy M., Courchesne-Loyer A., Croteau E., Tremblay S., Castellano A., Pifferi F., Bocti C., Paquet N. (2011). Brain fuel metabolism, aging, and Alzheimer’s disease. Nutrition.

[12] Magistretti P.J., Allaman I. (2015). A cellular perspective on brain energy metabolism and functional imaging. Neuron.

[13] Hebert A.S., Dittenhafer-Reed K.E., Yu W., Bailey D.J., Selen E.S., Boersma M.D., Carson J.J., Tonelli M., Balloon A.J., Higbee A.J. (2013). Calorie restriction and SIRT3 trigger global reprogramming of the mitochondrial protein acetylome. Mol. Cell.

[14] Han C., Someya S. (2013). Maintaining good hearing: Calorie restriction, Sirt3, and glutathione. Exp. Gerontol..

[15] Rose G., Dato S., Altomare K., Bellizzi D., Garasto S., Greco V., Passarino G., Feraco E., Mari V., Barbi C. (2003). Variability of the SIRT3 gene, human silent information regulator Sir2 homologue, and survivorship in the elderly. Exp. Gerontol..

[16] Albani D., Ateri E., Mazzuco S., Ghilardi A., Rodilossi S., Biella G., Ongaro F., Antuono P., Boldrini P., Di Giorgi E. (2014). Modulation of human longevity by SIRT3 single nucleotide polymorphisms in the prospective study “Treviso Longeva (TRELONG)”. Age.

[17] Barger J.L., Anderson R.M., Newton M.A., da Silva C., Vann J.A., Pugh T.D., Someya S., Prolla T.A., Weindruch R. (2015). A conserved transcriptional signature of delayed aging and reduced disease vulnerability is partially mediated by SIRT3. PLoS ONE.

[18] Brown K., Xie S., Qiu X., Mohrin M., Shin J., Liu Y., Zhang D., Scadden D.T., Chen D. (2013). SIRT3 reverses aging-associated degeneration. Cell Rep..

[19] Denu R.A. (2017). SIRT3 Enhances Mesenchymal Stem Cell Longevity and Differentiation. Oxid. Med. Cell. Longev..

[20] Diao Z., Ji Q., Wu Z., Zhang W., Cai Y., Wang Z., Hu J., Liu Z., Wang Q., Bi S. (2021). SIRT3 consolidates heterochromatin and counteracts senescence. Nucleic Acids Res..

[21] Zhang B., Cui S., Bai X., Zhuo L., Sun X., Hong Q., Fu B., Wang J., Chen X., Cai G. (2013). SIRT3 overexpression antagonizes high glucose accelerated cellular senescence in human diploid fibroblasts via the SIRT3-FOXO1 signaling pathway. Age.

[22] Onyango P., Celic I., McCaffery J.M., Boeke J.D., Feinberg A.P. (2002). SIRT3, a human SIR2 homologue, is an NAD-dependent deacetylase localized to mitochondria. Proc. Natl. Acad. Sci. USA.

[23] Hallows W.C., Albaugh B.N., Denu J.M. (2008). Where in the cell is SIRT3?—Functional localization of an NAD+-dependent protein deacetylase. Biochem J..

[24] Jin L., Galonek H., Israelian K., Choy W., Morrison M., Xia Y., Wang X., Xu Y., Yang Y., Smith J.J. (2009). Biochemical characterization, localization, and tissue distribution of the longer form of mouse SIRT3. Protein Sci..

[25] Cooper H.M., Spelbrink J.N. (2008). The human SIRT3 protein deacetylase is exclusively mitochondrial. Biochem. J..

[26] Scher M.B., Vaquero A., Reinberg D. (2007). SirT3 is a nuclear NAD+-dependent histone deacetylase that translocates to the mitochondria upon cellular stress. Genes Dev..

[27] Iwahara T., Bonasio R., Narendra V., Reinberg D. (2012). SIRT3 functions in the nucleus in the control of stress-related gene expression. Mol. Cell. Biol..

[28] Nakamura Y., Ogura M., Tanaka D., Inagaki N. (2008). Localization of mouse mitochondrial SIRT proteins: Shift of SIRT3 to nucleus by co-expression with SIRT5. Biochem. Biophys. Res. Commun..

[29] Hirschey M.D., Shimazu T., Huang J.Y., Verdin E. (2009). Acetylation of mitochondrial proteins. Methods Enzymol..

[30] Verdin E. (2015). NAD(+) in aging, metabolism, and neurodegeneration. Science.

[31] Sidorova-Darmos E., Sommer R., Eubanks J.H. (2018). The Role of SIRT3 in the Brain Under Physiological and Pathological Conditions. Front. Cell. Neurosci..

[32] White L.R., Edland S.D., Hemmy L.S., Montine K.S., Zarow C., Sonnen J.A., Uyehara-Lock J.H., Gelber R.P., Ross G.W., Petrovitch H. (2016). Neuropathologic comorbidity and cognitive impairment in the Nun and Honolulu-Asia Aging Studies. Neurology.

[33] Montine T.J., Sonnen J.A., Montine K.S., Crane P.K., Larson E.B. (2012). Adult Changes in Thought study: Dementia is an individually varying convergent syndrome with prevalent clinically silent diseases that may be modified by some commonly used therapeutics. Curr. Alzheimer Res..

[34] Kawas C.H., Kim R.C., Sonnen J.A., Bullain S.S., Trieu T., Corrada M.M. (2015). Multiple pathologies are common and related to dementia in the oldest-old: The 90+ Study. Neurology.

[35] Tyas S.L., Salazar J.C., Snowdon D.A., Desrosiers M.F., Riley K.P., Mendiondo M.S., Kryscio R.J. (2007). Transitions to mild cognitive impairments, dementia, and death: Findings from the Nun Study. Am. J. Epidemiol..

[36] Gelber R.P., Launer L.J., White L.R. (2012). The Honolulu-Asia Aging Study: Epidemiologic and neuropathologic research on cognitive impairment. Curr. Alzheimer Res..

[37] Gelber R.P., Ross G.W., Petrovitch H., Masaki K.H., Launer L.J., White L.R. (2013). Antihypertensive medication use and risk of cognitive impairment: The Honolulu-Asia Aging Study. Neurology.

[38] Osborne B., Reznick J., Wright L.E., Sinclair D.A., Cooney G.J., Turner N. (2022). Liver-specific overexpression of SIRT3 enhances oxidative metabolism, but does not impact metabolic defects induced by high fat feeding in mice. Biochem. Biophys. Res. Commun..

[39] Mukherjee S., Mo J., Paolella L.M., Perry C.E., Toth J., Hugo M.M., Chu Q., Tong Q., Chellappa K., Baur J.A. (2021). SIRT3 is required for liver regeneration but not for the beneficial effect of nicotinamide riboside. JCI Insight.

[40] Kendrick A.A., Choudhury M., Rahman S.M., McCurdy C.E., Friederich M., Van Hove J.L., Watson P.A., Birdsey N., Bao J., Gius D. (2011). Fatty liver is associated with reduced SIRT3 activity and mitochondrial protein hyperacetylation. Biochem. J..

[41] Chen M., Hui S., Lang H., Zhou M., Zhang Y., Kang C., Zeng X., Zhang Q., Yi L., Mi M. (2019). SIRT3 Deficiency Promotes High-Fat Diet-Induced Nonalcoholic Fatty Liver Disease in Correlation with Impaired Intestinal Permeability through Gut Microbial Dysbiosis. Mol. Nutr. Food Res..

[42] Pinteric M., Podgorski I.I., Popovic Hadzija M., Tartaro Bujak I., Tadijan A., Balog T., Sobocanec S. (2021). Chronic High Fat Diet Intake Impairs Hepatic Metabolic Parameters in Ovariectomized Sirt3 KO Mice. Int. J. Mol. Sci..

[43] Song Y.F., Zheng H., Luo Z., Hogstrand C., Bai Z.Y., Wei X.L. (2022). Dietary Choline Alleviates High-Fat Diet-Induced Hepatic Lipid Dysregulation via UPRmt Modulated by SIRT3-Mediated mtHSP70 Deacetylation. Int. J. Mol. Sci..

[44] Shi T., Fan G.Q., Xiao S.D. (2010). SIRT3 reduces lipid accumulation via AMPK activation in human hepatic cells. J. Dig. Dis..

[45] Bao J., Scott I., Lu Z., Pang L., Dimond C.C., Gius D., Sack M.N. (2010). SIRT3 is regulated by nutrient excess and modulates hepatic susceptibility to lipotoxicity. Free Radic. Biol. Med..

[46] Jing E., Emanuelli B., Hirschey M.D., Boucher J., Lee K.Y., Lombard D., Verdin E.M., Kahn C.R. (2011). Sirtuin-3 (Sirt3) regulates skeletal muscle metabolism and insulin signaling via altered mitochondrial oxidation and reactive oxygen species production. Proc. Natl. Acad. Sci. USA.

[47] Palacios O.M., Carmona J.J., Michan S., Chen K.Y., Manabe Y., Ward J.L., Goodyear L.J., Tong Q. (2009). Diet and exercise signals regulate SIRT3 and activate AMPK and PGC-1alpha in skeletal muscle. Aging.

[48] Jing E., O’Neill B.T., Rardin M.J., Kleinridders A., Ilkeyeva O.R., Ussar S., Bain J.R., Lee K.Y., Verdin E.M., Newgard C.B. (2013). Sirt3 regulates metabolic flexibility of skeletal muscle through reversible enzymatic deacetylation. Diabetes.

[49] Lantier L., Williams A.S., Williams I.M., Yang K.K., Bracy D.P., Goelzer M., James F.D., Gius D., Wasserman D.H. (2015). SIRT3 Is Crucial for Maintaining Skeletal Muscle Insulin Action and Protects Against Severe Insulin Resistance in High-Fat-Fed Mice. Diabetes.

[50] Boyle K.E., Newsom S.A., Janssen R.C., Lappas M., Friedman J.E. (2013). Skeletal muscle MnSOD, mitochondrial complex II, and SIRT3 enzyme activities are decreased in maternal obesity during human pregnancy and gestational diabetes mellitus. J. Clin. Endocrinol. Metab..

[51] Sundaresan N.R., Gupta M., Kim G., Rajamohan S.B., Isbatan A., Gupta M.P. (2009). Sirt3 blocks the cardiac hypertrophic response by augmenting Foxo3a-dependent antioxidant defense mechanisms in mice. J. Clin. Investig..

[52] Benigni A., Cassis P., Conti S., Perico L., Corna D., Cerullo D., Zentilin L., Zoja C., Perna A., Lionetti V. (2019). Sirt3 Deficiency Shortens Life Span and Impairs Cardiac Mitochondrial Function Rescued by Opa1 Gene Transfer. Antioxid. Redox Signal..

[53] He X., Zeng H., Chen S.T., Roman R.J., Aschner J.L., Didion S., Chen J.X. (2017). Endothelial specific SIRT3 deletion impairs glycolysis and angiogenesis and causes diastolic dysfunction. J. Mol. Cell. Cardiol..

[54] Chen T., Liu J., Li N., Wang S., Liu H., Li J., Zhang Y., Bu P. (2015). Mouse SIRT3 attenuates hypertrophy-related lipid accumulation in the heart through the deacetylation of LCAD. PLoS ONE.

[55] Koentges C., Pfeil K., Schnick T., Wiese S., Dahlbock R., Cimolai M.C., Meyer-Steenbuck M., Cenkerova K., Hoffmann M.M., Jaeger C. (2015). SIRT3 deficiency impairs mitochondrial and contractile function in the heart. Basic Res. Cardiol..

[56] Zeng H., Vaka V.R., He X., Booz G.W., Chen J.X. (2015). High-fat diet induces cardiac remodelling and dysfunction: Assessment of the role played by SIRT3 loss. J. Cell. Mol. Med..

[57] Guo X., Yan F., Li J., Zhang C., Su H., Bu P. (2020). SIRT3 Ablation Deteriorates Obesity-Related Cardiac Remodeling by Modulating ROS-NF-kappaB-MCP-1 Signaling Pathway. J. Cardiovasc. Pharmacol..

[58] Yu W., Qin J., Chen C., Fu Y., Wang W. (2018). Moderate calorie restriction attenuates age associated alterations and improves cardiac function by increasing SIRT1 and SIRT3 expression. Mol. Med. Rep..

[59] Bugga P., Alam M.J., Kumar R., Pal S., Chattopadyay N., Banerjee S.K. (2022). Sirt3 ameliorates mitochondrial dysfunction and oxidative stress through regulating mitochondrial biogenesis and dynamics in cardiomyoblast. Cell. Signal..

[60] He X., Zeng H., Chen J.X. (2019). Emerging role of SIRT3 in endothelial metabolism, angiogenesis, and cardiovascular disease. J. Cell. Physiol..

[61] Qiu C., Fratiglioni L. (2015). A major role for cardiovascular burden in age-related cognitive decline. Nat. Rev. Cardiol..

[62] Dikalov S., Dikalova A. (2022). Mitochondrial deacetylase Sirt3 in vascular dysfunction and hypertension. Curr. Opin. Nephrol. Hypertens..

[63] Yang L., Zhang J., Xing W., Zhang X., Xu J., Zhang H., Chen L., Ning X., Ji G., Li J. (2016). SIRT3 Deficiency Induces Endothelial Insulin Resistance and Blunts Endothelial-Dependent Vasorelaxation in Mice and Human with Obesity. Sci. Rep..

[64] Akhmedov A., Montecucco F., Costantino S., Vdovenko D., Schaub Clerigue A., Gaul D.S., Burger F., Roth A., Carbone F., Liberale L. (2020). Cardiomyocyte-Specific JunD Overexpression Increases Infarct Size Following Ischemia/Reperfusion Cardiac Injury by Downregulating Sirt3. Thromb. Haemost..

[65] Winnik S., Gaul D.S., Siciliani G., Lohmann C., Pasterk L., Calatayud N., Weber J., Eriksson U., Auwerx J., van Tits L.J. (2016). Mild endothelial dysfunction in Sirt3 knockout mice fed a high-cholesterol diet: Protective role of a novel C/EBP-beta-dependent feedback regulation of SOD2. Basic Res. Cardiol..

[66] Liu G., Cao M., Xu Y., Li Y. (2015). SIRT3 protects endothelial cells from high glucose-induced cytotoxicity. Int. J. Clin. Exp. Pathol..

[67] Chen T., Ma C., Fan G., Liu H., Lin X., Li J., Li N., Wang S., Zeng M., Zhang Y. (2021). SIRT3 protects endothelial cells from high glucose-induced senescence and dysfunction via the p53 pathway. Life Sci..

[68] Wang Y., Zhang X., Wang P., Shen Y., Yuan K., Li M., Liang W., Que H. (2019). Sirt3 overexpression alleviates hyperglycemia-induced vascular inflammation through regulating redox balance, cell survival, and AMPK-mediated mitochondrial homeostasis. J. Recept. Signal. Transduct. Res..

[69] Tyagi A., Mirita C., Shah I., Reddy P.H., Pugazhenthi S. (2021). Effects of Lipotoxicity in Brain Microvascular Endothelial Cells During Sirt3 Deficiency-Potential Role in Comorbid Alzheimer’s Disease. Front. Aging Neurosci..

[70] Zhao Z., Zhang X., Dai Y., Pan K., Deng Y., Meng Y., Xu T. (2019). PPAR-gamma promotes p38 MAP kinase-mediated endothelial cell permeability through activating Sirt3. BMC Neurol..

[71] Ahn B.H., Kim H.S., Song S., Lee I.H., Liu J., Vassilopoulos A., Deng C.X., Finkel T. (2008). A role for the mitochondrial deacetylase Sirt3 in regulating energy homeostasis. Proc. Natl. Acad. Sci. USA.

[72] Vassilopoulos A., Pennington J.D., Andresson T., Rees D.M., Bosley A.D., Fearnley I.M., Ham A., Flynn C.R., Hill S., Rose K.L. (2014). SIRT3 deacetylates ATP synthase F1 complex proteins in response to nutrient- and exercise-induced stress. Antioxid. Redox Signal..

[73] Cimen H., Han M.J., Yang Y., Tong Q., Koc H., Koc E.C. (2010). Regulation of succinate dehydrogenase activity by SIRT3 in mammalian mitochondria. Biochemistry.

[74] Yang Y., Cimen H., Han M.J., Shi T., Deng J.H., Koc H., Palacios O.M., Montier L., Bai Y., Tong Q. (2010). NAD+-dependent deacetylase SIRT3 regulates mitochondrial protein synthesis by deacetylation of the ribosomal protein MRPL10. J. Biol. Chem..

[75] Kwon S., Seok S., Yau P., Li X., Kemper B., Kemper J.K. (2017). Obesity and aging diminish sirtuin 1 (SIRT1)-mediated deacetylation of SIRT3, leading to hyperacetylation and decreased activity and stability of SIRT3. J. Biol. Chem..

[76] Kalsbeek M.J., Mulder L., Yi C.X. (2016). Microglia energy metabolism in metabolic disorder. Mol. Cell. Endocrinol..

[77] Norden D.M., Godbout J.P. (2013). Review: Microglia of the aged brain: Primed to be activated and resistant to regulation. Neuropathol. Appl. Neurobiol..

[78] Hickman S.E., Kingery N.D., Ohsumi T.K., Borowsky M.L., Wang L.C., Means T.K., El Khoury J. (2013). The microglial sensome revealed by direct RNA sequencing. Nat. Neurosci..

[79] Mhatre S.D., Tsai C.A., Rubin A.J., James M.L., Andreasson K.I. (2015). Microglial malfunction: The third rail in the development of Alzheimer’s disease. Trends Neurosci..

[80] Rardin M.J., Newman J.C., Held J.M., Cusack M.P., Sorensen D.J., Li B., Schilling B., Mooney S.D., Kahn C.R., Verdin E. (2013). Label-free quantitative proteomics of the lysine acetylome in mitochondria identifies substrates of SIRT3 in metabolic pathways. Proc. Natl. Acad. Sci. USA.

[81] Dittenhafer-Reed K.E., Richards A.L., Fan J., Smallegan M.J., Fotuhi Siahpirani A., Kemmerer Z.A., Prolla T.A., Roy S., Coon J.J., Denu J.M. (2015). SIRT3 mediates multi-tissue coupling for metabolic fuel switching. Cell. Metab..

[82] Tyagi A., Nguyen C.U., Chong T., Michel C.R., Fritz K.S., Reisdorph N., Knaub L., Reusch J.E.B., Pugazhenthi S. (2018). SIRT3 deficiency-induced mitochondrial dysfunction and inflammasome formation in the brain. Sci. Rep..

[83] Hallows W.C., Yu W., Smith B.C., Devries M.K., Ellinger J.J., Someya S., Shortreed M.R., Prolla T., Markley J.L., Smith L.M. (2011). Sirt3 promotes the urea cycle and fatty acid oxidation during dietary restriction. Mol. Cell..

[84] Bharathi S.S., Zhang Y., Mohsen A.W., Uppala R., Balasubramani M., Schreiber E., Uechi G., Beck M.E., Rardin M.J., Vockley J. (2013). Sirtuin 3 (SIRT3) protein regulates long-chain acyl-CoA dehydrogenase by deacetylating conserved lysines near the active site. J. Biol. Chem..

[85] Shimazu T., Hirschey M.D., Hua L., Dittenhafer-Reed K.E., Schwer B., Lombard D.B., Li Y., Bunkenborg J., Alt F.W., Denu J.M. (2010). SIRT3 deacetylates mitochondrial 3-hydroxy-3-methylglutaryl CoA synthase 2 and regulates ketone body production. Cell. Metab..

[86] Sorbara M.T., Girardin S.E. (2011). Mitochondrial ROS fuel the inflammasome. Cell. Res..

[87] Gurung P., Lukens J.R., Kanneganti T.D. (2015). Mitochondria: Diversity in the regulation of the NLRP3 inflammasome. Trends Mol. Med..

[88] Traba J., Sack M.N. (2017). The role of caloric load and mitochondrial homeostasis in the regulation of the NLRP3 inflammasome. Cell. Mol. Life Sci..

[89] Traba J., Geiger S.S., Kwarteng-Siaw M., Han K., Ra O.H., Siegel R.M., Gius D., Sack M.N. (2017). Prolonged fasting suppresses mitochondrial NLRP3 inflammasome assembly and activation via SIRT3-mediated activation of superoxide dismutase 2. J. Biol. Chem..

[90] Tyagi A., Mirita C., Taher N., Shah I., Moeller E., Tyagi A., Chong T., Pugazhenthi S. (2020). Metabolic syndrome exacerbates amyloid pathology in a comorbid Alzheimer’s mouse model. Biochim. Biophys. Acta Mol. Basis Dis..

[91] Dai S., Wei J., Zhang H., Luo P., Yang Y., Jiang X., Fei Z., Liang W., Jiang J., Li X. (2022). Intermittent fasting reduces neuroinflammation in intracerebral hemorrhage through the Sirt3/Nrf2/HO-1 pathway. J. Neuroinflamm..

[92] Qiu X., Brown K., Hirschey M.D., Verdin E., Chen D. (2010). Calorie restriction reduces oxidative stress by SIRT3-mediated SOD2 activation. Cell. Metab..

[93] Zeng L., Yang Y., Hu Y., Sun Y., Du Z., Xie Z., Zhou T., Kong W. (2014). Age-related decrease in the mitochondrial sirtuin deacetylase Sirt3 expression associated with ROS accumulation in the auditory cortex of the mimetic aging rat model. PLoS ONE.

[94] Xie L., Feng H., Li S., Meng G., Liu S., Tang X., Ma Y., Han Y., Xiao Y., Gu Y. (2016). SIRT3 Mediates the Antioxidant Effect of Hydrogen Sulfide in Endothelial Cells. Antioxid. Redox Signal..

[95] Yu W., Dittenhafer-Reed K.E., Denu J.M. (2012). SIRT3 protein deacetylates isocitrate dehydrogenase 2 (IDH2) and regulates mitochondrial redox status. J. Biol. Chem..

[96] Tseng A.H., Shieh S.S., Wang D.L. (2013). SIRT3 deacetylates FOXO3 to protect mitochondria against oxidative damage. Free Radic. Biol. Med..

[97] Wu W., Xu H., Wang Z., Mao Y., Yuan L., Luo W., Cui Z., Cui T., Wang X.L., Shen Y.H. (2015). PINK1-Parkin-Mediated Mitophagy Protects Mitochondrial Integrity and Prevents Metabolic Stress-Induced Endothelial Injury. PLoS ONE.

[98] Huang L., Yao T., Chen J., Zhang Z., Yang W., Gao X., Dan Y., He Y. (2022). Effect of Sirt3 on retinal pigment epithelial cells in high glucose through Foxo3a/ PINK1-Parkin pathway mediated mitophagy. Exp. Eye Res..

[99] Yu W., Gao B., Li N., Wang J., Qiu C., Zhang G., Liu M., Zhang R., Li C., Ji G. (2017). Sirt3 deficiency exacerbates diabetic cardiac dysfunction: Role of Foxo3A-Parkin-mediated mitophagy. Biochim. Biophys. Acta Mol. Basis Dis..

[100] Guo Y., Jia X., Cui Y., Song Y., Wang S., Geng Y., Li R., Gao W., Fu D. (2021). Sirt3-mediated mitophagy regulates AGEs-induced BMSCs senescence and senile osteoporosis. Redox Biol..

[101] Hu J., Liu T., Fu F., Cui Z., Lai Q., Zhang Y., Yu B., Liu F., Kou J., Li F. (2022). Omentin1 ameliorates myocardial ischemia-induced heart failure via SIRT3/FOXO3a-dependent mitochondrial dynamical homeostasis and mitophagy. J. Transl. Med..

[102] Xu S., Li L., Wu J., An S., Fang H., Han Y., Huang Q., Chen Z., Zeng Z. (2021). Melatonin Attenuates Sepsis-Induced Small-Intestine Injury by Upregulating SIRT3-Mediated Oxidative-Stress Inhibition, Mitochondrial Protection, and Autophagy Induction. Front. Immunol..

[103] Zhang T., Liu J., Tong Q., Lin L. (2020). SIRT3 Acts as a Positive Autophagy Regulator to Promote Lipid Mobilization in Adipocytes via Activating AMPK. Int. J. Mol. Sci..

[104] Wang H.N., Li J.L., Xu T., Yao H.Q., Chen G.H., Hu J. (2020). Effects of Sirt3autophagy and resveratrol activation on myocardial hypertrophy and energy metabolism. Mol. Med. Rep..

[105] Zhang M., Deng Y.N., Zhang J.Y., Liu J., Li Y.B., Su H., Qu Q.M. (2018). SIRT3 Protects Rotenone-induced Injury in SH-SY5Y Cells by Promoting Autophagy through the LKB1-AMPK-mTOR Pathway. Aging Dis..

[106] Wang Y., Chang J., Wang Z.Q., Li Y. (2021). Sirt3 promotes the autophagy of HK2 human proximal tubular epithelial cells via the inhibition of Notch1/Hes1 signaling. Mol. Med. Rep..

[107] Xin R., Xu Y., Long D., Mao G., Liao H., Zhang Z., Kang Y. (2022). Mitochonic Acid-5 Inhibits Reactive Oxygen Species Production and Improves Human Chondrocyte Survival by Upregulating SIRT3-Mediated, Parkin-dependent Mitophagy. Front. Pharmacol..

[108] Kim S.H., Lu H.F., Alano C.C. (2011). Neuronal Sirt3 protects against excitotoxic injury in mouse cortical neuron culture. PLoS ONE.

[109] Magnifico S., Saias L., Deleglise B., Duplus E., Kilinc D., Miquel M.C., Viovy J.L., Brugg B., Peyrin J.M. (2013). NAD+ acts on mitochondrial SirT3 to prevent axonal caspase activation and axonal degeneration. FASEB J..

[110] Dai S.H., Chen T., Wang Y.H., Zhu J., Luo P., Rao W., Yang Y.F., Fei Z., Jiang X.F. (2014). Sirt3 protects cortical neurons against oxidative stress via regulating mitochondrial Ca2+ and mitochondrial biogenesis. Int. J. Mol. Sci..

[111] Zhang X., Ren X., Zhang Q., Li Z., Ma S., Bao J., Li Z., Bai X., Zheng L., Zhang Z. (2016). PGC-1alpha/ERRalpha-Sirt3 Pathway Regulates DAergic Neuronal Death by Directly Deacetylating SOD2 and ATP Synthase beta. Antioxid. Redox Signal..

[112] Li X.H., Liu S.J., Liu X.Y., Zhao H.Y., Yang M.G., Xu D.X., Guo J., Li J.H., Li J.J. (2018). Expression of SIRT3 in various glial cell types in the periventricular white matter in the neonatal rat brain after hypoxia. Tissue Cell.

[113] Jiang D.Q., Wang Y., Li M.X., Ma Y.J., Wang Y. (2017). SIRT3 in Neural Stem Cells Attenuates Microglia Activation-Induced Oxidative Stress Injury Through Mitochondrial Pathway. Front. Cell. Neurosci..

[114] Jiang D.Q., Zang Q.M., Jiang L.L., Lu C.S., Zhao S.H., Xu L.C. (2022). SIRT3 expression alleviates microglia activationinduced dopaminergic neuron injury through the mitochondrial pathway. Exp. Ther. Med..

[115] Thangaraj A., Chivero E.T., Tripathi A., Singh S., Niu F., Guo M.L., Pillai P., Periyasamy P., Buch S. (2021). HIV TAT-mediated microglial senescence: Role of SIRT3-dependent mitochondrial oxidative stress. Redox Biol..

[116] Selkoe D.J. (2001). Alzheimer’s disease: Genes, proteins, and therapy. Physiol. Rev..

[117] Heneka M.T., Carson M.J., El Khoury J., Landreth G.E., Brosseron F., Feinstein D.L., Jacobs A.H., Wyss-Coray T., Vitorica J., Ransohoff R.M. (2015). Neuroinflammation in Alzheimer’s disease. Lancet Neurol..

[118] Wilkins H.M., Swerdlow R.H. (2016). Relationships Between Mitochondria and Neuroinflammation: Implications for Alzheimer’s Disease. Curr. Top. Med. Chem..

[119] Yang W., Zou Y., Zhang M., Zhao N., Tian Q., Gu M., Liu W., Shi R., Lu Y., Yu W. (2015). Mitochondrial Sirt3 Expression is Decreased in APP/PS1 Double Transgenic Mouse Model of Alzheimer’s Disease. Neurochem. Res..

[120] Lee J., Kim Y., Liu T., Hwang Y.J., Hyeon S.J., Im H., Lee K., Alvarez V.E., McKee A.C., Um S.J. (2018). SIRT3 deregulation is linked to mitochondrial dysfunction in Alzheimer’s disease. Aging Cell.

[121] Weir H.J., Murray T.K., Kehoe P.G., Love S., Verdin E.M., O’Neill M.J., Lane J.D., Balthasar N. (2012). CNS SIRT3 expression is altered by reactive oxygen species and in Alzheimer’s disease. PLoS ONE.

[122] Morris J.K., Honea R.A., Vidoni E.D., Swerdlow R.H., Burns J.M. (2014). Is Alzheimer’s disease a systemic disease?. Biochim. Biophys. Acta.

[123] Cheng A., Wang J., Ghena N., Zhao Q., Perone I., King T.M., Veech R.L., Gorospe M., Wan R., Mattson M.P. (2020). SIRT3 Haploinsufficiency Aggravates Loss of GABAergic Interneurons and Neuronal Network Hyperexcitability in an Alzheimer’s Disease Model. J. Neurosci..

[124] Liu Y., Cheng A., Li Y.J., Yang Y., Kishimoto Y., Zhang S., Wang Y., Wan R., Raefsky S.M., Lu D. (2019). SIRT3 mediates hippocampal synaptic adaptations to intermittent fasting and ameliorates deficits in APP mutant mice. Nat. Commun..

[125] Han P., Tang Z., Yin J., Maalouf M., Beach T.G., Reiman E.M., Shi J. (2014). Pituitary adenylate cyclase-activating polypeptide protects against beta-amyloid toxicity. Neurobiol. Aging.

[126] Ramesh S., Govindarajulu M., Lynd T., Briggs G., Adamek D., Jones E., Heiner J., Majrashi M., Moore T., Amin R. (2018). SIRT3 activator Honokiol attenuates beta-Amyloid by modulating amyloidogenic pathway. PLoS ONE.

[127] Nahalkova J. (2022). Focus on Molecular Functions of Anti-Aging Deacetylase SIRT3. Biochemistry.

[128] Tyagi A., Musa M., Labeikovsky W., Pugazhenthi S. (2022). Sirt3 deficiency induced down regulation of insulin degrading enzyme in comorbid Alzheimer’s disease with metabolic syndrome. Sci. Rep..

[129] Leal M.C., Magnani N., Villordo S., Buslje C.M., Evelson P., Castano E.M., Morelli L. (2013). Transcriptional regulation of insulin-degrading enzyme modulates mitochondrial amyloid beta (Abeta) peptide catabolism and functionality. J. Biol. Chem..

[130] Kurauti M.A., Freitas-Dias R., Ferreira S.M., Vettorazzi J.F., Nardelli T.R., Araujo H.N., Santos G.J., Carneiro E.M., Boschero A.C., Rezende L.F. (2016). Acute Exercise Improves Insulin Clearance and Increases the Expression of Insulin-Degrading Enzyme in the Liver and Skeletal Muscle of Swiss Mice. PLoS ONE.

[131] Shen Y., Wu Q., Shi J., Zhou S. (2020). Regulation of SIRT3 on mitochondrial functions and oxidative stress in Parkinson’s disease. Biomed. Pharmacother..

[132] Shi H., Deng H.X., Gius D., Schumacker P.T., Surmeier D.J., Ma Y.C. (2017). Sirt3 protects dopaminergic neurons from mitochondrial oxidative stress. Hum. Mol. Genet..

[133] Lee S., Jeon Y.M., Jo M., Kim H.J. (2021). Overexpression of SIRT3 Suppresses Oxidative Stress-induced Neurotoxicity and Mitochondrial Dysfunction in Dopaminergic Neuronal Cells. Exp. Neurobiol..

[134] Cui X.X., Li X., Dong S.Y., Guo Y.J., Liu T., Wu Y.C. (2017). SIRT3 deacetylated and increased citrate synthase activity in PD model. Biochem. Biophys. Res. Commun..

[135] Zhang J.Y., Deng Y.N., Zhang M., Su H., Qu Q.M. (2016). SIRT3 Acts as a Neuroprotective Agent in Rotenone-Induced Parkinson Cell Model. Neurochem. Res..

[136] Jiang D.Q., Ma Y.J., Wang Y., Lu H.X., Mao S.H., Zhao S.H. (2019). Microglia activation induces oxidative injury and decreases SIRT3 expression in dopaminergic neuronal cells. J. Neural Transm..

[137] Fu J., Jin J., Cichewicz R.H., Hageman S.A., Ellis T.K., Xiang L., Peng Q., Jiang M., Arbez N., Hotaling K. (2012). trans-(-)-epsilon-Viniferin increases mitochondrial sirtuin 3 (SIRT3), activates AMP-activated protein kinase (AMPK), and protects cells in models of Huntington Disease. J. Biol. Chem..

[138] Salamon A., Maszlag-Torok R., Veres G., Boros F.A., Vagvolgyi-Sumegi E., Somogyi A., Vecsei L., Klivenyi P., Zadori D. (2020). Cerebellar Predominant Increase in mRNA Expression Levels of Sirt1 and Sirt3 Isoforms in a Transgenic Mouse Model of Huntington’s Disease. Neurochem. Res..

[139] Naia L., Carmo C., Campesan S., Fao L., Cotton V.E., Valero J., Lopes C., Rosenstock T.R., Giorgini F., Rego A.C. (2021). Mitochondrial SIRT3 confers neuroprotection in Huntington’s disease by regulation of oxidative challenges and mitochondrial dynamics. Free Radic. Biol. Med..

[140] Song W., Song Y., Kincaid B., Bossy B., Bossy-Wetzel E. (2013). Mutant SOD1G93A triggers mitochondrial fragmentation in spinal cord motor neurons: Neuroprotection by SIRT3 and PGC-1alpha. Neurobiol. Dis..

[141] Hor J.H., Santosa M.M., Lim V.J.W., Ho B.X., Taylor A., Khong Z.J., Ravits J., Fan Y., Liou Y.C., Soh B.S. (2021). ALS motor neurons exhibit hallmark metabolic defects that are rescued by SIRT3 activation. Cell. Death Differ..

[142] Yang X., Geng K.Y., Zhang Y.S., Zhang J.F., Yang K., Shao J.X., Xia W.L. (2018). Sirt3 deficiency impairs neurovascular recovery in ischemic stroke. CNS Neurosci. Ther..

[143] Yang X., Geng K., Zhang J., Zhang Y., Shao J., Xia W. (2017). Sirt3 Mediates the Inhibitory Effect of Adjudin on Astrocyte Activation and Glial Scar Formation following Ischemic Stroke. Front. Pharmacol..

[144] Klimova N., Fearnow A., Long A., Kristian T. (2020). NAD(+) precursor modulates post-ischemic mitochondrial fragmentation and reactive oxygen species generation via SIRT3 dependent mechanisms. Exp. Neurol..

[145] Zhao H., Luo Y., Chen L., Zhang Z., Shen C., Li Y., Xu R. (2018). Sirt3 inhibits cerebral ischemia-reperfusion injury through normalizing Wnt/beta-catenin pathway and blocking mitochondrial fission. Cell Stress Chaperones.

[146] Liu L., Cao Q., Gao W., Li B., Xia Z., Zhao B. (2021). Melatonin protects against focal cerebral ischemia-reperfusion injury in diabetic mice by ameliorating mitochondrial impairments: Involvement of the Akt-SIRT3-SOD2 signaling pathway. Aging.

[147] Chen T., Liu W.B., Qian X., Xie K.L., Wang Y.H. (2021). The AMPAR antagonist perampanel protects the neurovascular unit against traumatic injury via regulating Sirt3. CNS Neurosci. Ther..

[148] Zhang S., Wu X., Wang J., Shi Y., Hu Q., Cui W., Bai H., Zhou J., Du Y., Han L. (2022). Adiponectin/AdiopR1 signaling prevents mitochondrial dysfunction and oxidative injury after traumatic brain injury in a SIRT3 dependent manner. Redox Biol..

[149] Huang W., Huang Y., Huang R.Q., Huang C.G., Wang W.H., Gu J.M., Dong Y. (2016). SIRT3 Expression Decreases with Reactive Oxygen Species Generation in Rat Cortical Neurons during Early Brain Injury Induced by Experimental Subarachnoid Hemorrhage. Biomed. Res. Int..

[150] Zeng H., He X., Tuo Q.H., Liao D.F., Zhang G.Q., Chen J.X. (2016). LPS causes pericyte loss and microvascular dysfunction via disruption of Sirt3/angiopoietins/Tie-2 and HIF-2alpha/Notch3 pathways. Sci. Rep..

[151] Zhou L., Pinho R., Gu Y., Radak Z. (2022). The Role of SIRT3 in Exercise and Aging. Cells.

[152] Fontana L., Partridge L. (2015). Promoting health and longevity through diet: From model organisms to humans. Cell.

[153] Shi Z., Li C., Yin Y., Yang Z., Xue H., Mu N., Wang Y., Liu M., Ma H. (2018). Aerobic Interval Training Regulated SIRT3 Attenuates High-Fat-Diet-Associated Cognitive Dysfunction. Biomed. Res. Int..

[154] Corpas R., Solana E., De la Rosa A., Sarroca S., Grinan-Ferre C., Oriol M., Corbella E., Rodriguez-Farre E., Vina J., Pallas M. (2019). Peripheral Maintenance of the Axis SIRT1-SIRT3 at Youth Level May Contribute to Brain Resilience in Middle-Aged Amateur Rugby Players. Front. Aging Neurosci..

[155] Brandauer J., Andersen M.A., Kellezi H., Risis S., Frosig C., Vienberg S.G., Treebak J.T. (2015). AMP-activated protein kinase controls exercise training- and AICAR-induced increases in SIRT3 and MnSOD. Front. Physiol..

[156] Camacho-Pereira J., Tarrago M.G., Chini C.C.S., Nin V., Escande C., Warner G.M., Puranik A.S., Schoon R.A., Reid J.M., Galina A. (2016). CD38 Dictates Age-Related NAD Decline and Mitochondrial Dysfunction through an SIRT3-Dependent Mechanism. Cell. Metab..

[157] Wang T., Zhang F., Peng W., Wang L., Zhang J., Dong W., Tian X., Ye C., Li Y., Gong Y. (2022). Overexpression of NMNAT3 improves mitochondrial function and enhances antioxidative stress capacity of bone marrow mesenchymal stem cells via the NAD+-Sirt3 pathway. Biosci. Rep..

[158] Brown K.D., Maqsood S., Huang J.Y., Pan Y., Harkcom W., Li W., Sauve A., Verdin E., Jaffrey S.R. (2014). Activation of SIRT3 by the NAD(+) precursor nicotinamide riboside protects from noise-induced hearing loss. Cell. Metab..

[159] Gong B., Pan Y., Vempati P., Zhao W., Knable L., Ho L., Wang J., Sastre M., Ono K., Sauve A.A. (2013). Nicotinamide riboside restores cognition through an upregulation of proliferator-activated receptor-gamma coactivator 1alpha regulated beta-secretase 1 degradation and mitochondrial gene expression in Alzheimer’s mouse models. Neurobiol. Aging.

[160] Martens C.R., Denman B.A., Mazzo M.R., Armstrong M.L., Reisdorph N., McQueen M.B., Chonchol M., Seals D.R. (2018). Chronic nicotinamide riboside supplementation is well-tolerated and elevates NAD(+) in healthy middle-aged and older adults. Nat. Commun..

[161] Conze D., Brenner C., Kruger C.L. (2019). Safety and Metabolism of Long-term Administration of NIAGEN (Nicotinamide Riboside Chloride) in a Randomized, Double-Blind, Placebo-controlled Clinical Trial of Healthy Overweight Adults. Sci. Rep..

[162] Zhang H., Ryu D., Wu Y., Gariani K., Wang X., Luan P., D’Amico D., Ropelle E.R., Lutolf M.P., Aebersold R. (2016). NAD(+) repletion improves mitochondrial and stem cell function and enhances life span in mice. Science.

[163] Wang X., Duan X., Yang G., Zhang X., Deng L., Zheng H., Deng C., Wen J., Wang N., Peng C. (2011). Honokiol crosses BBB and BCSFB, and inhibits brain tumor growth in rat 9L intracerebral gliosarcoma model and human U251 xenograft glioma model. PLoS ONE.

[164] Woodbury A., Yu S.P., Wei L., Garcia P. (2013). Neuro-modulating effects of honokiol: A review. Front. Neurol..

[165] Li H., Jia J., Wang W., Hou T., Tian Y., Wu Q., Xu L., Wei Y., Wang X. (2018). Honokiol Alleviates Cognitive Deficits of Alzheimer’s Disease (PS1V97L) Transgenic Mice by Activating Mitochondrial SIRT3. J. Alzheimers Dis..

[166] Wan R., Fan J., Song H., Sun W., Yin Y. (2022). Oxygen-Glucose Deprivation/Reperfusion-Induced Sirt3 Reduction Facilitated Neuronal Injuries in an Apoptosis-Dependent Manner During Prolonged Reperfusion. Neurochem. Res..

[167] Ye J.S., Chen L., Lu Y.Y., Lei S.Q., Peng M., Xia Z.Y. (2019). SIRT3 activator honokiol ameliorates surgery/anesthesia-induced cognitive decline in mice through anti-oxidative stress and anti-inflammatory in hippocampus. CNS Neurosci. Ther..

[168] Liu J., Zhang T., Zhu J., Ruan S., Li R., Guo B., Lin L. (2021). Honokiol attenuates lipotoxicity in hepatocytes via activating SIRT3-AMPK mediated lipophagy. Chin. Med..

[169] Liu J.X., Shen S.N., Tong Q., Wang Y.T., Lin L.G. (2018). Honokiol protects hepatocytes from oxidative injury through mitochondrial deacetylase SIRT3. Eur. J. Pharmacol..

[170] Pillai V.B., Samant S., Sundaresan N.R., Raghuraman H., Kim G., Bonner M.Y., Arbiser J.L., Walker D.I., Jones D.P., Gius D. (2015). Honokiol blocks and reverses cardiac hypertrophy in mice by activating mitochondrial Sirt3. Nat. Commun..

[171] Liao G., Zhao Z., Yang H., Li X. (2020). Honokiol ameliorates radiation-induced brain injury via the activation of SIRT3. J. Int. Med. Res..

[172] Bagul P.K., Katare P.B., Bugga P., Dinda A.K., Banerjee S.K. (2018). SIRT-3 Modulation by Resveratrol Improves Mitochondrial Oxidative Phosphorylation in Diabetic Heart through Deacetylation of TFAM. Cells.

[173] Bernal-Ramirez J., Silva-Platas C., Jerjes-Sanchez C., Ramos-Gonzalez M.R., Vazquez-Garza E., Chapoy-Villanueva H., Ramirez-Rivera A., Zarain-Herzberg A., Garcia N., Garcia-Rivas G. (2021). Resveratrol Prevents Right Ventricle Dysfunction, Calcium Mishandling, and Energetic Failure via SIRT3 Stimulation in Pulmonary Arterial Hypertension. Oxid. Med. Cell. Longev..

[174] Zhou X., Chen M., Zeng X., Yang J., Deng H., Yi L., Mi M.T. (2014). Resveratrol regulates mitochondrial reactive oxygen species homeostasis through Sirt3 signaling pathway in human vascular endothelial cells. Cell. Death Dis..

[175] Zheng M., Bai Y., Sun X., Fu R., Liu L., Liu M., Li Z., Huang X. (2022). Resveratrol Reestablishes Mitochondrial Quality Control in Myocardial Ischemia/Reperfusion Injury through Sirt1/Sirt3-Mfn2-Parkin-PGC-1alpha Pathway. Molecules.

[176] Yan W.J., Liu R.B., Wang L.K., Ma Y.B., Ding S.L., Deng F., Hu Z.Y., Wang D.B. (2018). Sirt3-Mediated Autophagy Contributes to Resveratrol-Induced Protection against ER Stress in HT22 Cells. Front. Neurosci..

[177] Wu J., Deng Z., Sun M., Zhang W., Yang Y., Zeng Z., Wu J., Zhang Q., Liu Y., Chen Z. (2020). Polydatin protects against lipopolysaccharide-induced endothelial barrier disruption via SIRT3 activation. Lab. Investig..

[178] Govindarajulu M., Ramesh S., Neel L., Fabbrini M., Buabeid M., Fujihashi A., Dwyer D., Lynd T., Shah K., Mohanakumar K.P. (2021). Nutraceutical based SIRT3 activators as therapeutic targets in Alzheimer’s disease. Neurochem. Int..

[179] Liu M., Zhang X., Wang Y. (2021). Curcumin Alleviates Abeta42-Induced Neuronal Metabolic Dysfunction via the Thrb/SIRT3 Axis and Improves Cognition in APPTG Mice. Neurochem. Res..

[180] Li Y., Wei X., Liu S.L., Zhao Y., Jin S., Yang X.Y. (2021). Salidroside protects cardiac function in mice with diabetic cardiomyopathy via activation of mitochondrial biogenesis and SIRT3. Phytother. Res..

[181] Han L., Li J., Li J., Pan C., Xiao Y., Lan X., Wang M. (2020). Activation of AMPK/Sirt3 pathway by phloretin reduces mitochondrial ROS in vascular endothelium by increasing the activity of MnSOD via deacetylation. Food Funct..

[182] Han W.M., Chen X.C., Li G.R., Wang Y. (2020). Acacetin Protects Against High Glucose-Induced Endothelial Cells Injury by Preserving Mitochondrial Function via Activating Sirt1/Sirt3/AMPK Signals. Front. Pharmacol..

[183] Liu D., Zhao L. (2022). Spinacetin alleviates doxorubicin-induced cardiotoxicity by initiating protective autophagy through SIRT3/AMPK/mTOR pathways. Phytomedicine.

[184] Duan W.J., Liang L., Pan M.H., Lu D.H., Wang T.M., Li S.B., Zhong H.B., Yang X.J., Cheng Y., Liu B. (2020). Theacrine, a purine alkaloid from kucha, protects against Parkinson’s disease through SIRT3 activation. Phytomedicine.

[185] Gao J.M., Zhang X., Shu G.T., Chen N.N., Zhang J.Y., Xu F., Li F., Liu Y.G., Wei Y., He Y.Q. (2022). Trilobatin rescues cognitive impairment of Alzheimer’s disease by targeting HMGB1 through mediating SIRT3/SOD2 signaling pathway. Acta Pharmacol. Sin..

[186] Ruankham W., Suwanjang W., Wongchitrat P., Prachayasittikul V., Prachayasittikul S., Phopin K. (2021). Sesamin and sesamol attenuate H2O2-induced oxidative stress on human neuronal cells via the SIRT1-SIRT3-FOXO3a signaling pathway. Nutr. Neurosci..

[187] Liu P., Zou D., Chen K., Zhou Q., Gao Y., Huang Y., Zhu J., Zhang Q., Mi M. (2016). Dihydromyricetin Improves Hypobaric Hypoxia-Induced Memory Impairment via Modulation of SIRT3 Signaling. Mol. Neurobiol..

[188] Li C., Miao X., Wang S., Liu Y., Sun J., Liu Q., Cai L., Wang Y. (2021). Elabela may regulate SIRT3-mediated inhibition of oxidative stress through Foxo3a deacetylation preventing diabetic-induced myocardial injury. J. Cell. Mol. Med..

[189] Du Y., Zhang J., Fang F., Wei X., Zhang H., Tan H., Zhang J. (2017). Metformin ameliorates hypoxia/reoxygenation-induced cardiomyocyte apoptosis based on the SIRT3 signaling pathway. Gene.

[190] Gao P., You M., Li L., Zhang Q., Fang X., Wei X., Zhou Q., Zhang H., Wang M., Lu Z. (2022). Salt-Induced Hepatic Inflammatory Memory Contributes to Cardiovascular Damage Through Epigenetic Modulation of SIRT3. Circulation.

